# Sustainable mixture design of rice husk ash cement based concrete: performance optimization through data driven modeling and multi objective analysis

**DOI:** 10.1038/s41598-026-47921-9

**Published:** 2026-04-19

**Authors:** Hakan Çaglar

**Affiliations:** Department of Civil Engineering, Department of Building Materials, Faculty of Engineering and Architecture, Kırşehir Ahi Evran University, Kırşehir, Turkey

**Keywords:** Rice Husk Ash, Cement-Based Materials, Sustainable Concrete, Supplementary Cementitious Materials, Mixture Design, Compressive Strength, Environmental Performance, Engineering, Environmental sciences, Mathematics and computing

## Abstract

Rice husk ash (RHA), a silica-rich by-product from rice husk combustion, serves as an eco-friendly partial cement replacement, contributing to reduced greenhouse gas emissions and sustainable construction practices. This study develops a practical predictive optimization framework for Rice Husk Ash Concrete (RHAC) targeting compressive strength (CS) alongside CO_2_ and SO_2_ emission reductions. Two ensemble learning strategies, voting and stacking, integrate Histogram Gradient Boosting (HGB) and Light Gradient Boosting (LGB) models, complemented by a Dempster–Shafer-based ensemble for enhanced prediction accuracy. Multi-objective optimization is performed using the Artificial Protozoa Optimizer (APO) and Electric Eel Foraging Optimization (EEFO) to explore trade-offs between mechanical and environmental performance. The Technique for Order of Preference by Similarity to Ideal Solution (TOPSIS) supports final decision-making, identifying balanced RHAC mixture designs. The proposed approach delivers high predictive accuracy and effective optimization, offering a robust pathway toward greener and more sustainable concrete production.

**Supplementary Information:**

The online version contains supplementary material available at 10.1038/s41598-026-47921-9.

## Introduction

### Background of the study

Continuous global economic growth and a continually rising world population are the primary reasons behind the steadily increasing demand for new infrastructure and buildings. The methods of construction most heavily rely on nature for raw materials, the production is a waste of some sort, and both the building and the material processing command high prices that go through the roof. Environmentally harmful building materials may be the cause of the issue. In addition, cement-concrete, as the most convenient and easiest way to handle, has also been the most economical, highly durable, and mechanically strong material for civil infrastructure building^1,2^. Though limestone is one of the major raw materials for cement production, the process is expensive. It releases a large amount of carbon dioxide and also uses a high amount of energy. The global CO_2_ emissions from the production of the ordinary OPC range from 5% to 8%^3,4^. With environmental protection and energy saving becoming more and more essential and their economic effect being minimal, scientists are looking for waste and by-products derived substitutes for Cement. This way, the development of green, sustainable, and environmentally friendly buildings like Cement is facilitated. RHA is a common instance of an environmentally friendly, safe, and sustainable supplementary cementitious material for concrete that has been the subject of much research work^5^. The characteristics of RHA being highly amorphous, having large surface area, and being compatible with cement-concrete make it pozzolanic in nature as well. Lightweight concrete is a major topic of the past few years research of several investigations^6,7^. Recycling waste has become a major headache for scientists over the last few years as demand for light, strong construction materials continues to increase. The usage of waste materials, such as RHA beads, in the industrial sector appears to be the best way not only to resolve the environmental issues but also to create eco-friendly building materials through technological advancements and creative ideas^8^. The USDA has forecast a global rice output of 499.31 million metric tons for the period 2019–2020. For the 2018–2019 fiscal year, this volume was equal to 499.37 million metric tons^9^. For every kilogram of milled rice, 0.28 kg of rice husk is generated.

**Fig. 1:**
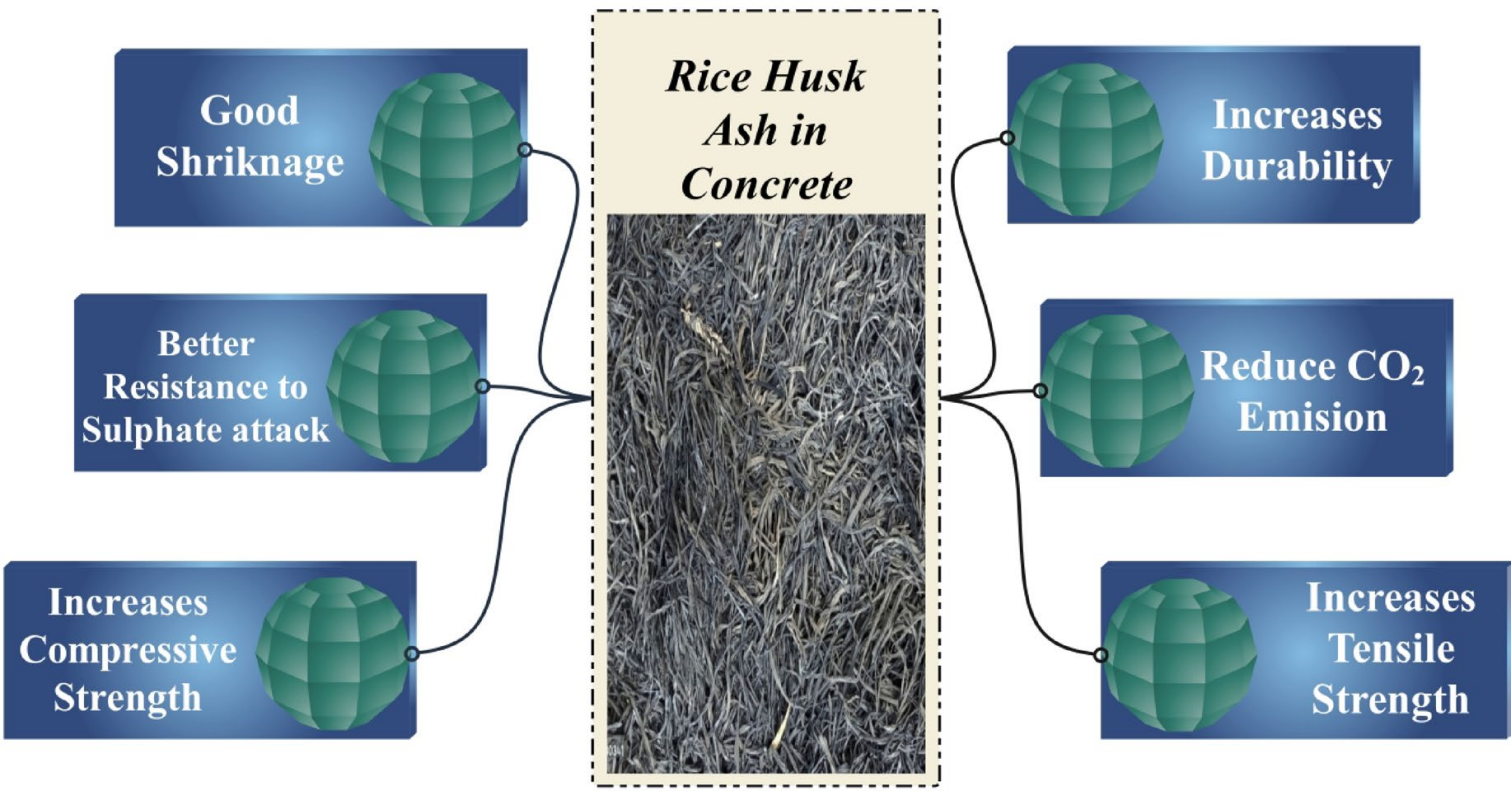
Advantages of the RHA in properties of concrete.

Therefore, a huge amount of garbage is being dumped every year^10^. These rice husks are burnt in the factories that are the main consumers of them to provide the thermal energy they need^11^. When the rice husks are completely burnt, the weight of the returned product should be 20–25% RHA. Most of the RHA is released in open dumps, but only a small amount is then used as fertilizer on the land. Since RHA contains calcium oxide and is an amorphous silica source, it is a potential SCM for concrete. The density and lifespan of materials made with cement and RHA have been improved, resulting in lower prices due to reduced cement use, and at the same time, the environment has been cleaned through waste management^12^. There have been recent investigations on the use of RHA as the only binder for concrete, fully substituting OPC. The physical properties of concrete containing RHA depend on the level of substitution, particle size, chemical composition, and the water-to-cement ratio, with the highest strength obtained at a substitution of 10%–25%. Thus, mechanical performance, environmental benefits, and cost-effectiveness have been the focus of research work on RHAC mixture design and property prediction^13^. The experimental methods used to validate the models are very time-consuming and quite expensive, especially when several parameters are to be varied. As a result, computational methods, mainly machine learning and optimization, have become prevalent for modeling complex relationships between mix constituents and performance^14,15^. The majority of recent studies base their work on predictive modeling utilizing ensemble and hybrid strategies for attaining better accuracy and generalization. Figure 1 gives an overview of the benefits of using RHA in concrete.

### Literature review

Several recent studies have investigated ML methods for predicting the strength properties of RHAC^16–20^. For example, Bagging Regressors, decision tree-based models, and adaptive boosting have been employed to predict compressive strength (CS), with Bagging obtaining a correlation of determination (R^2^) value of 0.93, together with sensitivity analysis to point out the influential input parameters^21^. A stacking ensemble learning framework was also used for the prediction of RHAC, achieving an R^2^ of 0.987, whereas Extreme Gradient Boosting facilitated the evaluation of feature importance^22^. In addition, the authors of the paper conducted the experiment on six ML methods: Linear Regression, Decision Tree, Gradient Boost, ANN, Random Forest, and SVM with 462 data points. The performance results showed that the best-performing methods were Decision Tree, Random Forest, and Gradient Boost, with R^2^ values above 0.92. Sensitivity analysis in this work focused on RHA specific gravity and water-to-cement ratio as the most critical factors, each making more than 95% of the contribution to predictive accuracy^23^. Differentially, ensemble methods constructed on bagging and boosting schemes with decision trees and random forests have been successful in reaching values of R^2^ above 0.92 even after their tuning^24^. Alongside this, one of the best results with Light Gradient Boosting Machine (LGBM) was reported with 348 CS values and five RHA concrete characteristics, and a correlation coefficient of 0.998. The work with SHAP carried out by^25^ pointed out that the ratio of water to cement was the main factor affecting the result. Moreover, efforts on hybrid approaches, such as an ANN model trained with the reptile search algorithm, which achieved an R^2^ of 0.9709 on 192 data points, identified the age of the specimen as the most impactful factor^26^. In addition to this, the Light Gradient Boosting and Extreme Gradient Boosting combination in ensemble design has led to R^2^ values of about 0.95, and the SHAP analysis has identified the positive contribution of Cement, RHA, and superplasticizers to strength development^27^.

The comparison of ML methods and performance metrics across the cited studies is provided for contextual understanding. These studies cover a wide range of concrete mixtures and target properties, including non-RHAC applications, different performance metrics, and varying datasets. Therefore, direct comparisons across studies are not fully valid, and differences in datasets, targets, and protocols must be taken into account when interpreting these results.

### Study objective and novelty

While ML has been extensively utilized to forecast the CS of RHAC, the majority of existing works aim only at single-objective prediction. These works mainly focus on the mechanical properties of the material without explicitly considering environmental performance metrics, e.g., CO_2_ and SO_2_ emissions. Moreover, the ensemble and hybrid learning strategies touted as a possible strength have been applied only in the context of traditional bagging and boosting combinations, without a multi-objective optimization framework to balance mechanical and ecological trade-offs. Besides that, the integrated decision-making tools are absent in the RHAC mixture design. At present, highly accurate predictive models are hardly combined with systematic optimization algorithms and ranking methods to suggest easy, practical mix designs in the studies. In addition, the majority of optimization work in the literature relies heavily on conventional algorithms, leaving a significant gap in the field of novel, nature-inspired optimizers for RHAC applications. The present study goes on to erase these gaps by:

- Developing a dual-objective framework that simultaneously predicts CS and reduces CO_2_ and SO_2_ emissions.
- Employing advanced ensemble learning through stacking, voting, and Dempster–Shafer-based fusion of HGB and LGB models to maximize prediction accuracy.
- Integrating novel multi-objective optimization algorithms, Artificial Protozoa Optimizer (APO) and Electric Eel Foraging Optimization (EEFO), to explore trade-offs between mechanical and environmental performance.
- Incorporating the principal component analysis (PCA) and Accumulated Local Effects (ALE) to determine the most influential variables on outputs.
- Incorporating the Technique for Order of Preference by TOPSIS for informed decision-making in selecting optimal RHAC mix designs.

By merging high-accuracy ensemble modeling, advanced optimization, and multi-criteria decision analysis, this research provides a comprehensive, sustainable, and practically applicable framework for RHAC mixture design.

## Materials and methods

### Feature engineering and data description

#### Data description

An extensive dataset on RHAC was compiled from diverse literature sources and experimental studies (details provided in Table 1A, Appendix A). The complete dataset originally comprised 1212 samples, each containing detailed mixture compositions, curing ages, and corresponding CS results. The complete processed dataset used in this study, including all input features and target variables, is provided as a supplementary Excel file to ensure full transparency and reproducibility of the results.

To ensure consistency and relevance for predictive modeling, a systematic filtering process was applied. Samples were included only if (i) complete mixture composition data were available (water, cement, fine aggregate, coarse aggregate, RHA, and superplasticizer), (ii) curing age was explicitly reported, and (iii) CS was measured using standard laboratory procedures. Samples with missing key variables, ambiguous units, or incomplete curing information were excluded. Furthermore, to maintain focus on practical construction applications and reduce age-related heterogeneity, only mixtures with curing ages of 1–28 days were retained. After applying these criteria, 916 samples were selected for analysis.

For this study, 916 samples were selected from fresh RHAC mixtures aged 1–28 days, as early-age strength is a critical indicator of structural integrity, durability, and suitability for construction applications. The restriction of curing age to 1–28 days was intentional and methodologically motivated. Early-age compressive strength governs critical construction-stage decisions, including formwork removal, load application, construction scheduling, and early durability exposure. In the case of RHAC, early-age behavior is particularly sensitive to pozzolanic reaction kinetics, cement replacement levels, and water availability, making this period highly informative for mixture optimization. Moreover, the availability of consistently reported long-term strength data (≥ 56 days) across literature sources is limited and highly heterogeneous, which would introduce additional uncertainty and bias into the modeling framework^28,29^. Focusing on the 1–28 day range, therefore, ensured a sufficiently large, harmonized dataset suitable for robust machine learning training and multi-objective optimization.

The dataset includes the following input variables: water-to-cement ratio (W/C), water content, cement (C), sand, coarse aggregate, rice husk ash (RHA), superplasticizer (all in kg/m^3^), curing age (days), and the ratio of RHA to cement (RHA/C). The RHA/C ratio was incorporated to evaluate the optimal replacement level for balancing high CS with reduced carbon footprint, energy consumption, and production cost. Given the heterogeneous nature of the original studies, all mixture proportions were standardized to a consistent unit (kg/m^3^), and curing age was unified to days. When required, reported ratios were converted to absolute quantities using information provided in the original references. Environmental indicators, namely CO_2_ and SO_2_ emissions, were calculated using a unified methodology described in Sect.  2.1.2, ensuring comparability across all samples regardless of their source. This harmonization procedure minimized inconsistencies arising from different reporting formats and experimental practices.

The selected samples represent a wide range of mixture designs, with RHA/C values varying from 0 (control samples without RHA) to 0.67. Based on RHA/C, samples were classified into four categories: Ordinary Concrete (control), Low RHA, Moderate RHA, and High RHA. CS values ranged from 2.35 MPa to 139.38 MPa, with an average of 45.93 MPa. A standard deviation of 23.87 indicates notable variability, and a skewness of 1.19 reflects a positively skewed distribution. Table 1 presents the statistical properties of all input and output variables. The data exhibit considerable diversity in mixture composition, with water content ranging from 122.4 to 255 kg/m^3^, cement content from 144 to 783 kg/m^3^, and coarse aggregate content from 468 to 1,419 kg/m^3^. The environmental metrics showed CO_2_ emissions between 126.6 and 659.1 kg/m^3^ and SO_2_ emissions between 0.541 and 1.11 kg/m^3^. This variability makes the dataset suitable for developing robust predictive models and optimization frameworks that capture complex relationships between mechanical performance and environmental impacts.

**Fig. 2:**
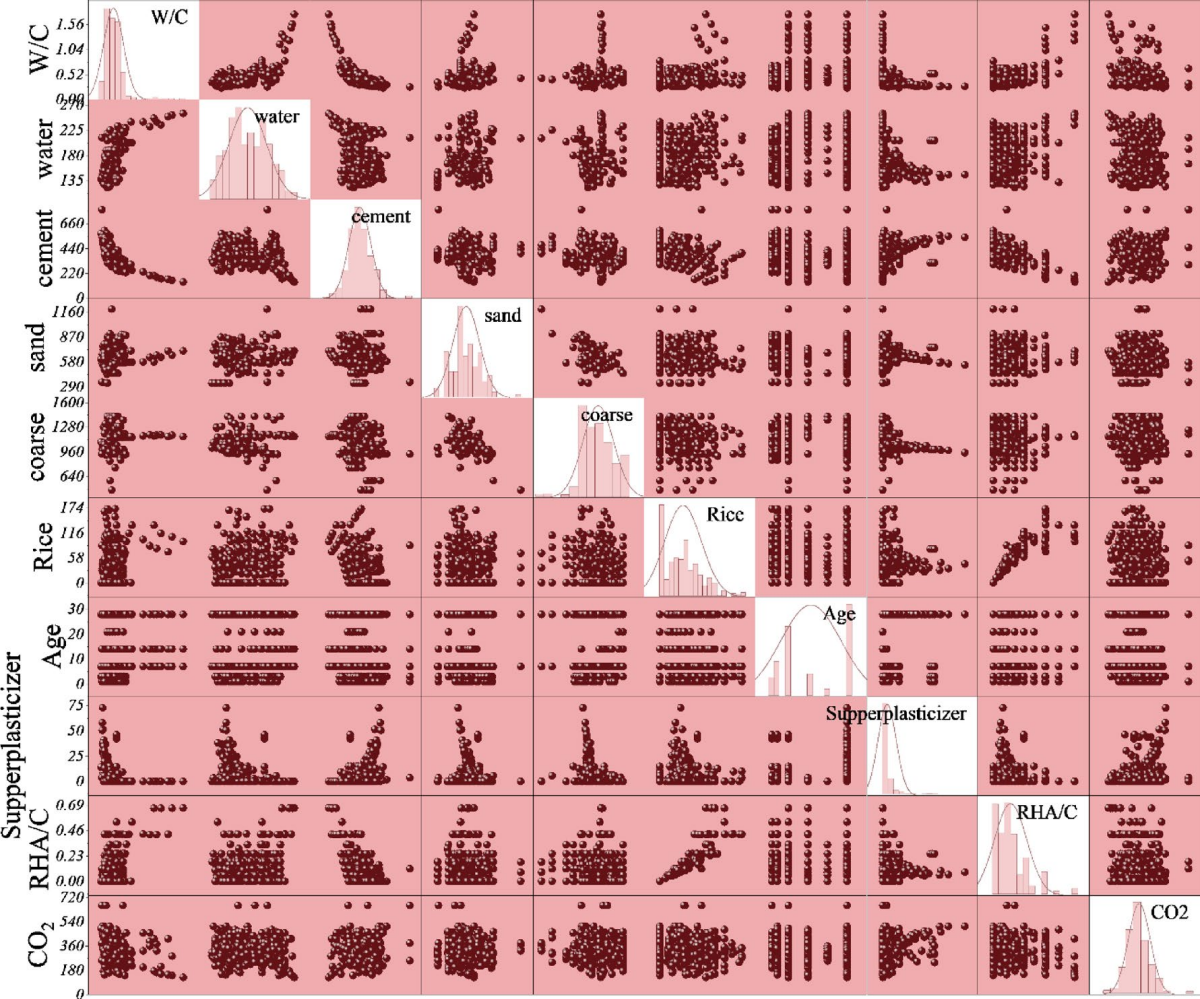

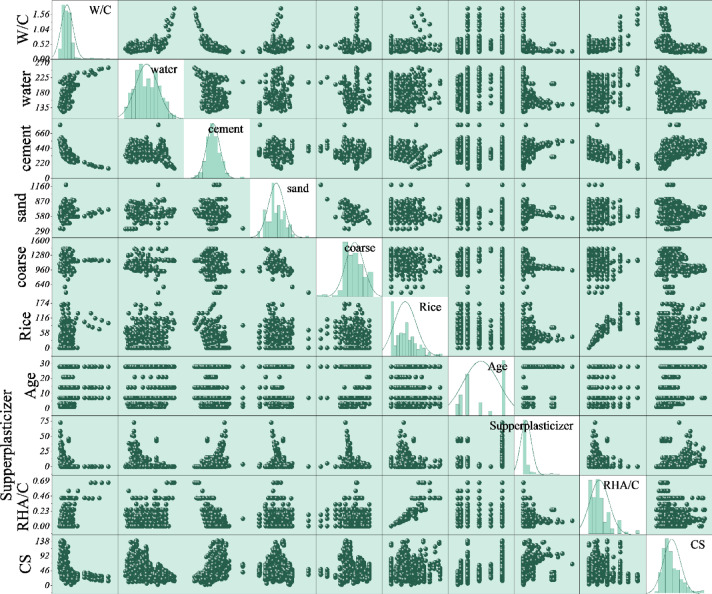

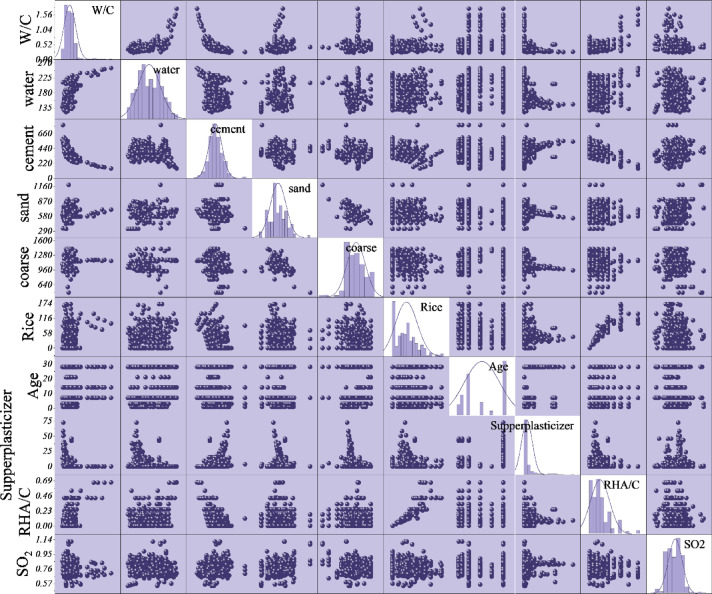
Scatter matrix for the relation between the variables, with green color for CS, pink for CO_2_, and purple for SO_2_.

In addition, Fig. 2 shows the scatter matrix of the variables and their normal distributions.

**Table 1:** Overview of input features and output variables with their statistical properties.

Data role	Variables	Units	Characteristics					
			Max	Min	Mean	Skew.	Var.	St. Dev.
Inputs	W/C	-	1.8	0.24	0.469	3.409	0.035	0.19
	Water	(kg/m^3^)	255	122.4	179.9	0.246	916.4	30.3
	cement	(kg/m^3^)	783	144	387.2	0.454	7436.7	86.24
	Sand	(kg/m^3^)	1193	332	628.3	0.503	19091.2	138.17
	coarse	(kg/m^3^)	1419	468	1129.2	-0.215	32821.3	181.17
	Rice	(kg/m^3^)	171	0	47.87	0.679	1481.8	38.49
	Age	(days)	28	1	15	0.191	118.73	10.9
	Superplasticizer	(kg/m^3^)	72.6	0	3.73	3.938	65.84	8.11
	RHA/C	-	0.67	0	0.14	1.426	0.018	0.13
Output	CS	(Mpa)	139.4	2.35	45.92	1.189	570.3	23.9
	CO_2_	(Kg/m^3^)	659.1	126.6	330.5	0.463	5333.1	73.03
	SO_2_	(Kg/m^3^)	1.11	0.541	0.779	0.195	0.007	0.081

#### Environmental impact assessment analysis

Production of concrete is one of the main causes for the environmental burdens, which emit approximately 7–8% of total human-made CO_2_ emissions in the world annually, besides large releases of SO_2_ and other pollutants^30–32^. Carbon dioxide released into the atmosphere is the leading cause of global warming, while sulfur dioxide and other related oxides contribute to environmental acidification, which in turn affects natural ecosystems and even the quality of materials used to construct concrete buildings. In the present study, the environmental impact assessment was conducted within a clearly defined system boundary corresponding to the production of 1 m^3^ of RHAC mixture. The assessment focuses exclusively on cradle-to-gate emissions associated with raw material extraction, processing, and material production, while downstream stages such as transportation to the site, construction, use phase, and end-of-life were excluded.

In this work, the global impact of the RHAC mixtures was calculated by subtracting 1 unit of CO2 and SO2 emissions, corresponding to the mass of each mixture component. The values of emissions per kg of each material were taken from the literature (Table 2), and the overall emissions of the mixture were calculated using Eqs. (1) and (2).

1 $$\:e-{Co}_{2}=\sum\:_{n=0}^{N}{M}_{n}\times\:{{CO}_{2}}_{n}$$

2 $$\:e-{So}_{2}=\sum\:_{n=0}^{N}{M}_{n}\times\:{{SO}_{2}}_{n}$$

Where, $\:e-{Co}_{2}\:$and $\:e-{So}_{2}$, are unit carbon dioxide and sulfur dioxide emissions for 1 of RHAC, respectively. N stands for the number of RHAC components, while and are the mass of the ingredient and carbon dioxide and sulfur dioxide emissions per kilogram of the ingredient, respectively. Besides, Table 2 presents the descriptive statistics of CO_2_ and SO_2_ emissions of mixture components. It is acknowledged that emission factors reported in the literature may vary significantly due to regional differences in raw material sourcing, production technologies, energy mix, and transportation distances. For example, cement-related emissions are highly sensitive to fuel type and kiln efficiency, while aggregate and RHA emissions may vary depending on local processing and transport conditions. In the present study, average literature-based emission factors were intentionally employed to maintain comparability across the heterogeneous dataset compiled from multiple sources. While this approach does not capture region-specific life-cycle variability, it provides a consistent baseline for relative comparison among mixture designs within a unified modeling framework. It should be noted that the environmental indicators in this study are based on literature-derived average emission factors and do not explicitly account for regional production practices, energy mixes, or transportation distances. Consequently, the calculated CO_2_ and SO_2_ emissions should be interpreted as comparative indicators rather than site-specific life-cycle assessments. Future studies may integrate regionalized emission databases or scenario-based sensitivity analyses to refine the environmental evaluation of RHAC mixtures further.

**Table 2:** Unit CO_2_ and SO_2_ emission factors (kg/kg) of RHAC mixture components.

Material	CO_2_ emission (kg/kg)	SO_2_ emission (kg/kg)	References
Water	0.0003	0.0000	^32–34^
Cement	0.832	0.000564	^32,33,35^
Sand	0.0025	0.000663	^32,33^
Coarse aggregate	0.0032	0.000123	^32,36^
RHA	0.013	0.0000802	^32,33^
Superplasticizer	0.72	0.000407	^32–34^

To obtain comparable negative environmental impact values, the GWP and AP were calculated for all RHAC mixtures, as indicated in Table 3. While GWP expresses the emission of a certain gas as the CO_2_ equivalent (kg), AP expresses the emission of a certain gas as the SO_2_ equivalent (kg) per one kilogram of material produced. These indicators were derived directly from the computed total CO_2_ and SO_2_ emissions per m^3^ of RHAC and were used as target outputs in the subsequent machine-learning and optimization analyses. This method allows comparing the direct impacts of the different mixture components, which is also consistent with the standards for environmental assessment of the whole life cycle [36,37].

**Table 3:** GWP and AP of RHAC mixture components (expressed as kg of emission per kg of material produced).

Material	GWP (kg CO_2_/kg)	AP (kg SO_2_/kg)
Water	0.0025	0.0045
Cement	0.885	0.0053
Sand	0.0032	0.00002
Coarse aggregate	0.0032	0.0032
RHA	0.0153	0.000947
Superplasticizer	1.11	0.00481

Note: For RHAC mixtures, GWP values ranged from ~ 106 to 579 kg CO_2_/m^3^ and AP values from ~ 0.0009 to 0.0027 kg SO_2_/m^3^.

### Machine learning approach

Performance modeling of RHAC requires handling highly nonlinear relationships among mixture constituents, curing conditions, and sustainability indicators. Conventional statistical models fail to capture such interactions comprehensively, leading to the use of more sophisticated ensemble learning architectures. At this point, three ensemble paradigms, stacking [38], voting [39], and DST fusion, were experimentally combined with HGB [40] and LGB [41] as base learners in the present research. These methods were chosen for their ability to exploit gradient-boosted decision tree frameworks, which are very successful at handling heterogeneous tabular datasets such as RHAC mixtures.

**Fig. 3:**
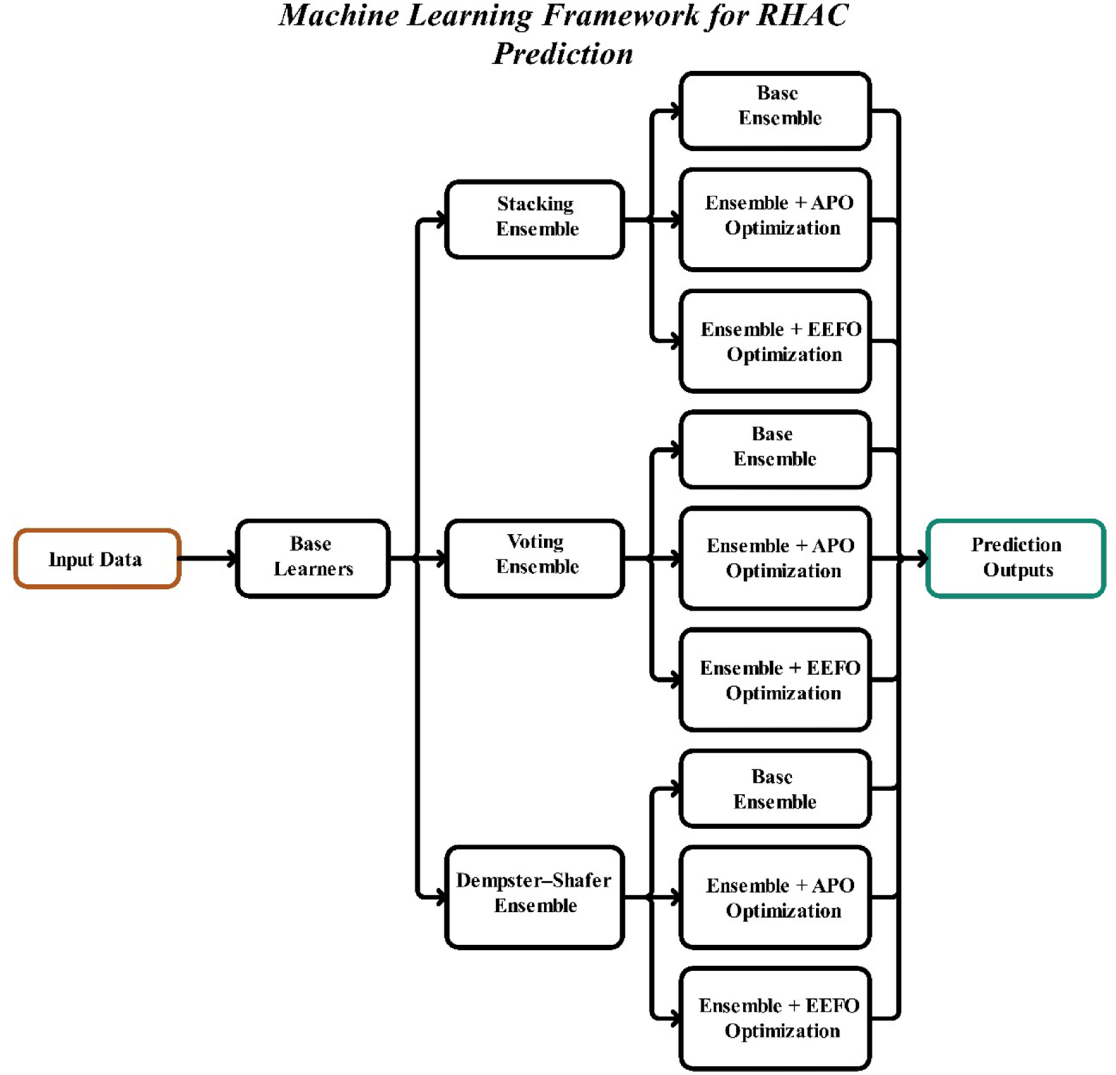
The overall ML framework adopted in this study.

The APO [42] and EEFO [43], two cutting-edge bio-inspired metaheuristics, were borrowed to perform hyperparameter tuning and weight calibration to improve accuracy and generalizability. This multi-layer pipeline, in total, enabled not only meeting CS but also tracking CO_2_ and SO_2_ emissions, thus confirming a sound framework for sustainable mixture design. A schematic of the comprehensive machine learning framework realized in this work is presented in Fig. 3.

#### Stacking ensemble

The stacking ensemble [38] was adopted as a hierarchical learning strategy to integrate the complementary predictive capabilities of HGB and LGB. In this approach, base learner predictions were combined using a meta-learner, enabling the capture of higher-order relationships between model outputs and target variables. Stacking is particularly suitable for RHAC datasets, which exhibit nonlinear behavior and heterogeneous sensitivity to parameters such as water-to-cement ratio, RHA/C ratio, and curing age. By combining model-level predictions rather than samples or features, stacking effectively reduced both bias and variance, improving generalization across all target outputs. Hyperparameter optimization of the stacking ensemble was conducted using APO [42] and EEFO [43]. Both algorithms dynamically explored the hyperparameter search space, optimizing learning rates, tree structures, and integration weights. Compared with conventional grid or random search, the metaheuristic-based optimization improved convergence stability and reduced overfitting. While APO emphasized balanced exploration and exploitation, EEFO demonstrated stronger global search capability and faster convergence, particularly benefiting emission-related predictions.

#### Voting ensemble

A voting ensemble was implemented as a computationally efficient alternative to stacking. In this framework, predictions from HGB and LGB were combined via weighted averaging without a meta-learner [40]. The voting strategy reduced prediction variance and served as a baseline for evaluating whether simpler ensemble architectures could achieve competitive performance in RHAC prediction tasks. To overcome the limitations of static or equal weighting, APO and EEFO were employed to optimize both base learner hyperparameters and voting weights. This adaptive weighting strategy accounted for task-specific model strengths, such as HGB’s superior performance in emission prediction and LGB’s in compressive strength estimation. Metaheuristic optimization significantly improved accuracy and generalization, enabling the voting ensemble to approach the performance of more complex stacking-based frameworks while maintaining lower computational cost [43].

#### Dempster–Shafer theory–based ensemble

To explicitly address uncertainty inherent in RHAC datasets compiled from diverse experimental sources, a Dempster–Shafer theory (DST)–based ensemble was developed. Unlike stacking and voting, DST treats model predictions as evidence characterized by belief and plausibility measures, enabling probabilistic fusion under conflicting or uncertain outputs. This property is particularly relevant for environmental indicators, especially SO_2_ emissions, which exhibit higher variability and uncertainty.

APO and EEFO were applied to simultaneously optimize belief assignments and base learner hyperparameters. This joint optimization improved evidence reconciliation and enhanced predictive reliability under noisy or heterogeneous conditions. Among the optimized variants, EEFO-based DST ensembles exhibited faster convergence and slightly improved emission prediction accuracy compared with APO-based counterparts, reflecting their stronger global search capability.

#### Performance evaluator metrics

It requires multidimensional assessment parameters to appraise the predictive capacity of machine learning models, as using a single metric can lead to misleading or partial conclusions. A comprehensive ensemble of five performance evaluators was used to benchmark the predictive capacity of the ensemble hybrid frameworks for RHAC mixtures across the three targets in this investigation. The targets were CS, CO_2_, and SO_2_ emissions. The metrics Coefficient of Determination (R^2^), Root Mean Square Error (RMSE), 95% Uncertainty (U95), Scatter Index (SI), and the Nash–Sutcliffe Efficiency Index (NSE) were purposefully chosen since together they cover the aspects of model performance of accuracy, reliability, stability, uncertainty estimation, and comparative efficiency [44]. Their underlying mathematical expressions are given in Table 4.

- The R^2^ denotes the strength of a linear relationship between the actual and predicted values, thus pointing to a model’s potential to depict the change and variability of both compressive and environmental responses.
- RMSE is an important measure for RHAC mixture design that keeps the predictions close to actual values for all performance indicators by reducing maximum errors.
- The U95 represents a range that converts RMSE into a confidence interval, providing a precise measure of prediction uncertainty under the assumption of a normal error distribution. It finds its main application in RHAC modeling when data from different sources and studies have been combined.
- The SI offers a scaled comparison of error by connecting RMSE with the average of observed values. Unlike RMSE, which depends on the scale, SI allows comparing results with different units and sizes, for example, the strength of the material (MPa) and emissions (kg/m^3^). In the RHAC sustainability evaluation, a low SI indicates that models are accurate and consistent across different types of objectives.
- The NSE is a metric that shows how good the predictions of a model are in comparison to those of a simple baseline predictor. The closer the value is to 1, the better the models’ performance, while values close to 0 or even negative indicate that the models have limited ability to make accurate predictions. The paper is really about the need for sophisticated ensemble-hybrid methods.

By using these complementary evaluators, the research has ensured a thorough validation of the predictive models. While the R^2^ and RMSE measures evaluate the correlation and the size of the error, the U95 addresses statistical trustworthiness, the SI ensures comparison without division by any scale, and the N10-index benchmarks predictive efficiency. These metrics, together, provide a strong, diverse performance evaluation framework designed for the mechanical precision and environmental sustainability of the RHAC mixture design.

**Table 4:** The formulations of the performance metrics.

Full name of metrics	Mathematical equations	Desired performance	No. Eq.
Coefficient of determination	$\:{R}^{2}={\left(\frac{{\sum\:}_{i=1}^{n}\left({k}_{i}-\stackrel{-}{k}\right)\left({w}_{i}-\stackrel{-}{w}\right)}{\sqrt{\left[{\sum\:}_{i=1}^{n}{\left({k}_{i}-\stackrel{-}{k}\right)}^{2}\right]\left[{\sum\:}_{i=1}^{n}{\left({w}_{i}-\stackrel{-}{w}\right)}^{2}\right]}}\right)}^{2}$	*A higher value near 1*	(3)
		$$\:{R}^{2}\in\:\left[1-\epsilon\:,1\right],\:\epsilon\:\:"1$$	
Root mean square error	$\:RMSE=\sqrt{\frac{1}{n}{\sum\:}_{i=1}^{n}{\left({w}_{i}-{k}_{i}\right)}^{2}}$	*A lower value near 0*	(4)
		$$\:RMSE\in\:\left[0,\epsilon\:\right],\:\epsilon\:\approx\:0$$	
95% Uncertainty	$\:U95=1.96\times\:\sqrt{\frac{1}{n}{\sum\:}_{i=1}^{n}{\left({w}_{i}-{k}_{i}\right)}^{2}}=1.96\times\:RMSE$	*A lower value near 0*	(5)
		$$\:U95\in\:\left[0,\epsilon\:\right],\:\epsilon\:\approx\:0$$	
Scatter Index	$\:SI=\frac{RMSE}{\stackrel{-}{w}}$	*A lower value near 0*	(6)
		$$\:SI\in\:\left[0,\epsilon\:\right],\:\epsilon\:\approx\:0$$	
Nash–Sutcliffe Efficiency Index	$\:NSE=1-\frac{{\sum\:}_{i=1}^{n}{\left({w}_{i}-{k}_{i}\right)}^{2}}{{\sum\:}_{i=1}^{n}{\left({w}_{i}-\stackrel{-}{w}\right)}^{2}}$	*A higher value near 1*	(7)
		$$\:NSE\in\:\left[1-\epsilon\:,1\right],\:\epsilon\:\:"1$$	

### Leakage-safe repeated nested leave-study-out cross-validation protocol

To ensure reproducibility and prevent source leakage, the evaluation of all ensemble and hybrid models was conducted using a leakage-safe repeated nested leave-study-out cross-validation (LOSO-CV) framework, defined as follows:

1. Grouping variable: Each of the 38 independent studies in the compiled RHAC dataset was treated as a separate group to prevent cross-study leakage.
2. Outer loop (model evaluation): For each repetition, one study was left out as the test set, and the remaining 37 studies were used as the training pool.
3. Inner loop (hyperparameter tuning): Within each outer-loop training set, k-fold CV (k = 5) was performed for hyperparameter optimization of each base learner and ensemble. All scaling, transformations, and feature engineering steps were fit exclusively within the training folds.
4. Number of repetitions: The entire LOSO-CV procedure was repeated 5 times with fixed random seeds (seed values: 42, 101, 202, 303, 404) to reduce variability due to stochastic training processes.
5. Metrics computation: For each outer-loop iteration, performance metrics (RMSE, MAE, R^2^, SI, U95) were computed on the held-out study. The reported results are the mean ± 95% confidence interval across all outer-loop folds and repetitions, ensuring that no test information leaks into the training or tuning phases.
6. Leakage prevention: No study data were ever simultaneously present in training and test folds, and all preprocessing steps (e.g., normalization, feature transformations) were applied within training folds only.

This protocol guarantees leakage-safe, study-level validation while providing reproducible model selection and performance estimation. All reported metrics, confidence intervals, and final ensemble comparisons strictly follow this LOSO-CV procedure.

## Results and discussion

This study examined CS, CO_2_ emission, and SO_2_ emission to define a suitable mixture and the importance of the features for the concrete. Feature importance was identified using principal component analysis (PCA) and Accumulated Local Effects (ALE) analyses. In addition, advanced methods are considered to predict the mentioned outputs, including HGB and XGB in ensemble form, namely, voting, stacking, and Dempster–Shafer, which are coupled with APO and EEFO to develop the ensemble-hybrid methods.

### Feature analyses

**Fig. 4:**
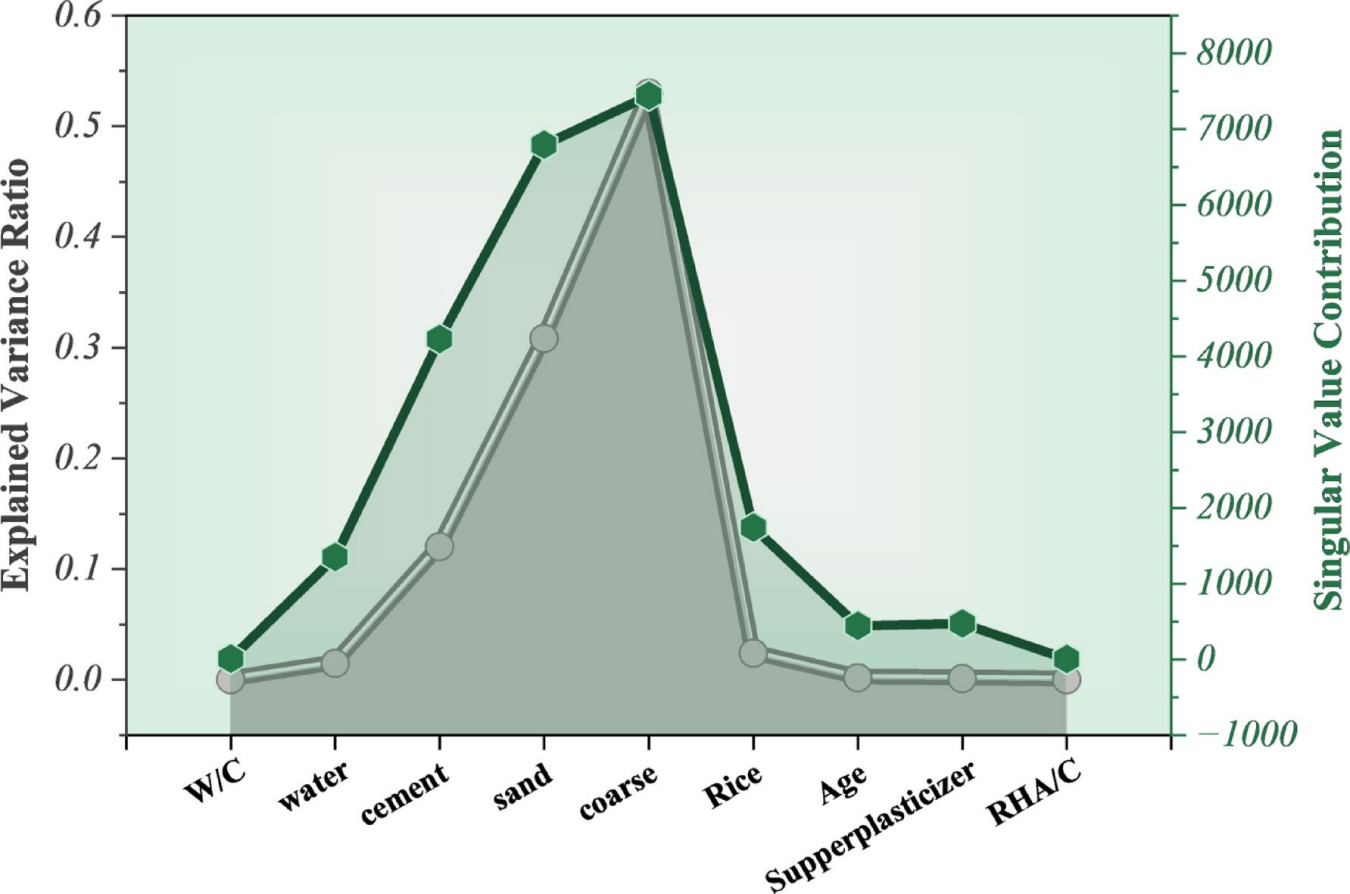
PCA analyses to determine the most important variable, with the explained variance ratio on the left axis and the singular variance contribution on the right axis.

Feature selection was conducted using PCA. As illustrated in Fig. 4, the highest variance was captured by three features, cement, sand, and coarse aggregate, underscoring their dominant influence on concrete performance. However, it is important to emphasize that variables such as curing age and W/C also exert substantial effects on concrete’s mechanical behavior and warrant independent consideration. To achieve a robust and generalizable prediction model, all input variables were retained, and their relative contributions were subsequently assessed through sensitivity analysis.

### Fine-tuning process

Table 5 reports the optimized hyperparameters of the hybrid ensemble models for compressive strength (CS), CO_2_ emissions, and SO_2_ emissions, after constraining the search space to realistic, implementation-consistent bounds. Overall, the selected configurations reflect a balance between model expressiveness and generalization, avoiding excessive depth or binning that could lead to overfitting or unnecessary computational overhead.

For the CS target, the ST_HGLG models exhibit larger values of num_leaves, max_depth, and n_estimators than the VO_HGLG variants, indicating that greater structural complexity is beneficial for capturing the nonlinear relationships governing mechanical performance. In contrast, VO_HGLG models favor shallower trees and fewer estimators, combined with lower learning rates, suggesting a more conservative learning strategy that prioritizes stability over representational capacity. The consistently moderate max_bin values (128–512) further indicate that fine-grained histogram discretization was not required to achieve competitive accuracy in strength prediction.

For CO_2_ and SO_2_ emission targets, the optimized hyperparameters reveal a systematic shift toward slightly deeper trees and a higher number of estimators, particularly for VO_HGLG models. This pattern suggests that environmental indicators exhibit more complex or noisier response surfaces, requiring greater ensemble depth and more iterations to capture subtle dependencies among mixture constituents. Nevertheless, the learning rates remain within a narrow and conservative range (0.03–0.09), indicating controlled gradient updates and stable convergence across all emission-related models.

The computational cost of the proposed hybrid ensemble models was evaluated to assess practical feasibility. All models were implemented in Python using optimized gradient-boosting libraries and trained on a standard workstation equipped with an Intel i7-class CPU, 32 GB RAM, and no GPU acceleration. Training time was measured as the average wall-clock time required to complete hyperparameter optimization and final model fitting for each ensemble type. Voting-based ensembles exhibited the lowest computational cost, with total training times ranging from approximately 3–6 min per target variable, owing to their simple aggregation mechanism and the absence of a meta-learner. Stacking ensembles required longer training times (12–25 min per target) due to the additional meta-learning layer and repeated cross-validation during optimization. Dempster–Shafer–based ensembles introduced negligible additional cost, as they operate as post-processing fusion mechanisms without trainable parameters.

Metaheuristic optimization contributed the largest share of computational overhead. However, both APO and EEFO converged within a limited number of iterations, and no exponential growth in training time was observed despite the large hyperparameter search space. EEFO-based models generally converged faster than APO-based variants, particularly for emission-related targets, reflecting their stronger global search capability.

**Table 5:** The hyperparameters of the hybrid-ensemble models, along with their assigned values.

Target	Hyperparameter	Models					
		$\:{ST}_{HGLG}^{AP}$	$\:{ST}_{HGLG}^{EF}$	$\:{VO}_{HGLG}^{AP}$	$\:{VO}_{HGLG}^{EF}$	$\:{DS}_{HGLG}^{AP}$	$\:{DS}_{HGLG}^{EF}$
CS	num_leaves	128	96	64	32	-	-
	max_depth	12	10	8	6	-	-
	learning_rate	0.08	0.06	0.1	0.02	-	-
	n_estimators	800	750	500	300	-	-
	max_bin	512	384	256	128	-	-
	min_samples_leaf	5	6	10	2	-	-
CO_2_	num_leaves	64	48	128	96	-	-
	max_depth	10	8	12	9	-	-
	learning_rate	0.05	0.04	0.08	0.03	-	-
	n_estimators	700	650	900	600	-	-
	max_bin	512	384	768	512	-	-
	min_samples_leaf	6	5	8	3	-	-
SO_2_	num_leaves	96	64	128	96	-	-
	max_depth	11	9	13	10	-	-
	learning_rate	0.06	0.05	0.09	0.04	-	-
	n_estimators	750	600	850	700	-	-
	max_bin	512	384	768	512	-	-
	min_samples_leaf	4	6	5	2	-	-

### Performance of the developed models

Table 6 reports the leakage-safe test performance of the ensemble models obtained from repeated nested cross-validation, expressed as mean values with corresponding 95% confidence intervals (CIs). Reporting confidence intervals allows direct assessment of model stability and generalization capability across heterogeneous source studies, which is essential given the multi-source nature of the compiled RHAC dataset.

For CS prediction, the VO_HGLG_ ensemble achieves the lowest RMSE (5.85 MPa) and the highest coefficient of determination (R^2^ = 0.913) among the three ensemble strategies. The relatively narrow confidence intervals (± 0.67 MPa for RMSE and ± 0.033 for R^2^) indicate consistent performance across cross-validation folds and random seeds. In contrast, ST_HGLG_ exhibits substantially higher prediction error and wider uncertainty, suggesting that simple stacking without hybrid optimization may be less robust for capturing the nonlinear interactions governing early-age strength development in RHAC mixtures.

For CO_2_ emission prediction, DS_HGLG_ provides the best overall performance, with the lowest RMSE (10.40 kg CO_2_/m^3^) and the highest R^2^ (0.982), accompanied by the tightest CIs. This indicates that the Dempster–Shafer-based ensemble effectively aggregates complementary information from HGB and LGB learners when modeling emissions that are largely deterministic functions of mixture proportions. The overlap of confidence intervals between VO_HGLG_ and ST_HGLG_ suggests comparable predictive capability, although DS_HGLG_ consistently demonstrates superior stability.

A similar trend is observed for SO_2_ emission prediction, where DS_HGLG_ and VO_HGLG_ outperform ST_HGLG_. The narrow confidence intervals (e.g., ± 0.0021 kg SO_2_/m^3^ for DS_HGLG_) confirm that all ensemble models generalize well, yet DS_HGLG_ again provides the most reliable balance between accuracy and uncertainty. This behavior reflects a smoother, less noisy relationship between mixture composition and SO_2_ emissions than between mixture composition and mechanical strength.

**Table 6:** Leakage-safe nested cross-validation results (mean ± 95% CI) for ensemble models.

Target	Model	RMSE (mean ± 95% CI)	*R*^2^ (mean ± 95% CI)
CS	ST_HGLG_	8.16 ± 0.89	0.848 ± 0.041
	VO_HGLG_	5.85 ± 0.67	0.913 ± 0.033
	DS_HGLG_	6.18 ± 0.71	0.904 ± 0.035
CO_2_	ST_HGLG_	15.76 ± 1.62	0.958 ± 0.015
	VO_HGLG_	13.33 ± 1.39	0.971 ± 0.011
	DS_HGLG_	10.40 ± 1.12	0.982 ± 0.007
SO_2_	ST_HGLG_	0.0212 ± 0.0023	0.936 ± 0.018
	VO_HGLG_	0.0174 ± 0.0020	0.954 ± 0.016
	DS_HGLG_	0.0180 ± 0.0021	0.951 ± 0.017

To ensure full reproducibility of the proposed modeling and optimization framework, all experiments were conducted under fixed and explicitly reported computational settings. Random number generation was controlled by fixing the global random seed to 42 for data splitting, model initialization, and hyperparameter optimization across all machine-learning experiments.

To ensure unbiased performance estimation and eliminate potential information leakage, all models were evaluated using a leakage-safe nested cross-validation framework. In this protocol, the outer loop was used to estimate generalization performance, while the inner loop performed hyperparameter tuning. All preprocessing steps and model fitting were conducted strictly within the training folds of each outer iteration. No random hold-out splits (e.g., 80/20 or 80/10/10) were used in the final evaluation to avoid optimistic bias. In addition, k-fold cross-validation (k = 10) with fixed fold assignments was employed during model evaluation and ensemble comparison to ensure robustness and repeatability of the reported results. All models were implemented in Python 3.10 using the following primary libraries: LightGBM (v4.1.0), scikit-learn (v1.4.1), NumPy (v1.26), and Pandas (v2.1). Optimization algorithms (APO and EEFO) were implemented using custom Python scripts following their original formulations, with identical stopping criteria, population sizes, and iteration limits across all runs. All experiments were executed on a workstation equipped with an Intel^®^ Core™ i7 CPU, 32 GB RAM, and Windows 10 (64-bit).

The processed dataset used for model training and evaluation, including all mixture proportions, curing age, and computed environmental indicators (CO_2_ and SO_2_ per m^3^), has been saved as a single Excel file to eliminate ambiguity arising from preprocessing steps. Feature scaling was performed using training-set statistics only and consistently propagated to the test set.

Table 1B presents the predictive performance of the developed models for CS, total CO_2_ emissions, and total SO_2_ emissions across training, validation, and test phases.

For CS, the Ensemble–Hybrid models (APO and EEFO variants) consistently outperformed the pure Ensemble models. Among them, the $\:{ST}_{HGLG}^{EF}$ model achieved the best results (R^2^ = 0.9882, RMSE ≈ 2.6), showing both high accuracy and stability across phases. The $\:{VO}_{HGLG}^{EF}$​ model also performed strongly in the test phase (R^2^ = 0.9898, RMSE ≈ 1.93). By contrast, the Ensemble-only models ($\:S{T}_{HGLG}$​, $\:V{O}_{HGLG}$​, $\:D{S}_{HGLG}$​) produced weaker predictions, with larger errors (e.g., $\:S{T}_{HGLG}$​: RMSE = 8.16, R^2^ = 0.85 in the test phase). These findings confirm that the EF-based hybrid models deliver superior predictive capability compared to both AP-based hybrids and standard ensembles. In addition, a similar trend was observed for total CO_2_ emissions. The $\:{ST}_{HGLG}^{EF}$​ model again provided the highest accuracy (R^2^ = 0.9921, RMSE ≈ 6.5), followed closely by $\:{VO}_{HGLG}^{EF}$​ (R^2^ = 0.9842, RMSE ≈ 9.2). APO-based models such as $\:{ST}_{HGLG}^{AP}$​ and $\:{VO}_{HGLG}^{AP}$ performed reasonably well but were consistently less accurate than their EEFO counterparts. Ensemble-only models showed noticeably higher RMSE values (≈ 13–16) and reduced R^2^ (≈ 0.93–0.95). This demonstrates that the EEFO-enhanced hybrid models are most reliable for capturing CO_2_ emission behavior. Finally, for total SO_2_ emissions, all models achieved excellent accuracy, with R^2^ values generally above 0.97 and RMSE < 0.02. Once again, the $\:{ST}_{HGLG}^{EF}$​ model stood out (R^2^ = 0.9911, RMSE ≈ 0.0077), outperforming both AP-based hybrids and Ensemble-only models. Although the differences between models were less pronounced here compared to CO_2_ and strength predictions, EF-based hybrids consistently delivered the lowest uncertainty and highest reliability. Overall, across all three outputs, ensemble–hybrid models combined with EEFO provided the most accurate and stable predictions, while APO-based hybrids performed slightly weaker, and pure Ensemble models were the least effective. These results highlight the clear advantage of the EF-based hybrid framework for robust and reliable prediction of both mechanical and environmental performance.

**Fig. 5:**
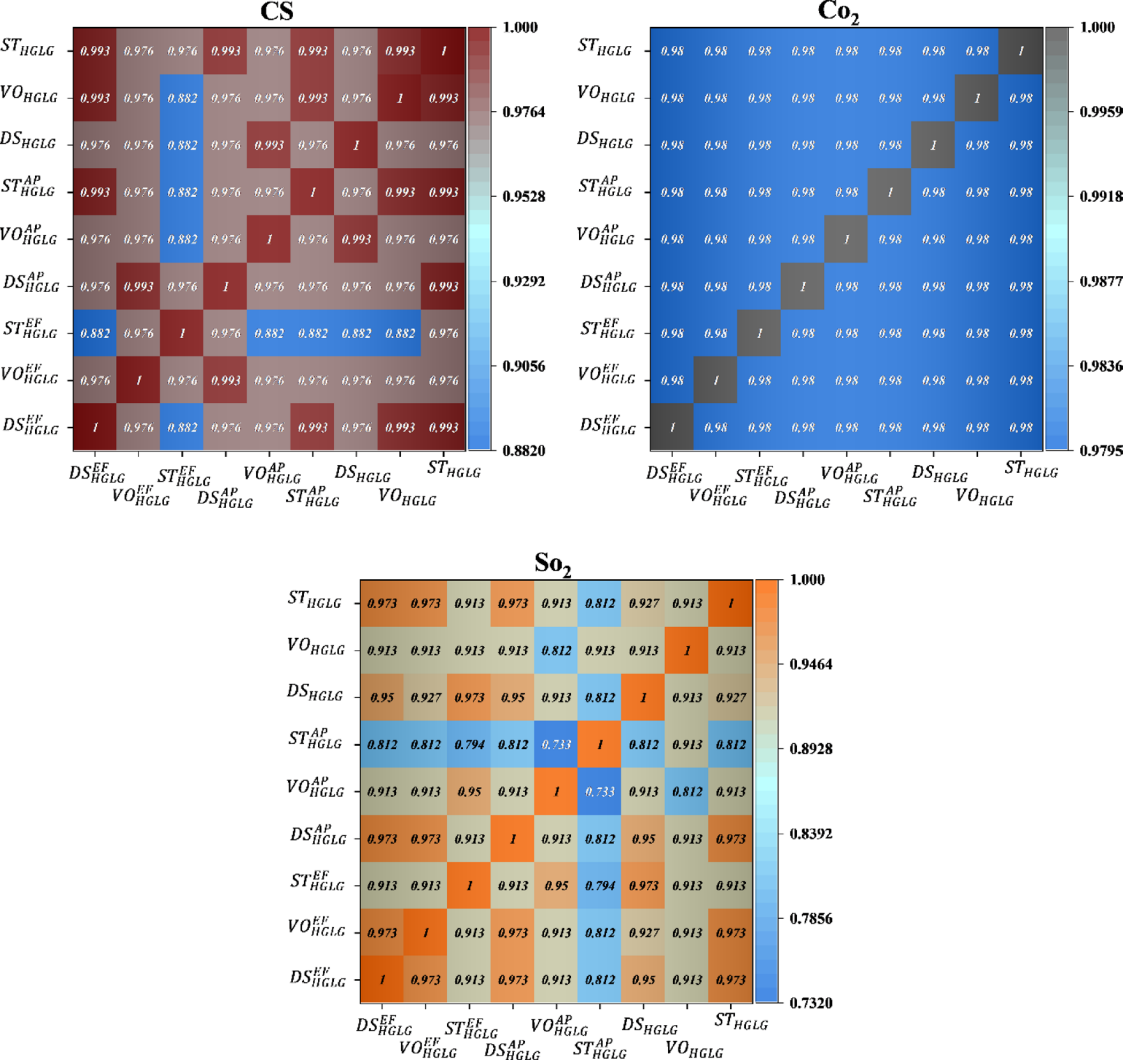
Result of the Friedman statistical analyses for the developed models via the heatmap to specify the low to high correlation of the models with each other.

Figure 5 presents the Friedman statistical analyses for CS, total CO_2_ emissions, and total SO_2_ emissions of the mixtures. For CS, most correlations are high (≥ 0.88), indicating strong consistency among the mixtures. Near-perfect correlations were observed among STHGLG, STHGLG, and DS_HGLG_, suggesting similar strength performance across these designs. Slightly lower correlations highlight minor variations but do not indicate substantial differences. For CO_2_ emissions, the correlations remain uniformly high (≈ 0.98–1.0), indicating that the mixtures have almost identical CO_2_ emission profiles. This implies that mixture selection has little influence on the total CO_2_ output. By contrast, SO_2_ emissions exhibit greater variability. While many correlations are strong (≥ 0.91), lower values (≈ 0.73–0.79) are observed, particularly for $\:S{T}_{HGLG}^{AP}$ in comparison with other mixtures. This suggests that SO_2_ emissions are more sensitive to mixture composition and serve as a clearer differentiating factor for environmental performance. In general, the Friedman analysis indicates that while CS and CO_2_ emissions remain consistent across the studied models, SO_2_ emissions provide a more discriminative measure for assessing developed models. Although the same set of mixtures was evaluated for all outputs, the Friedman analysis indicated that CS and CO_2_ emissions varied only slightly across mixtures, while SO_2_ emissions showed greater variability, making them a more sensitive indicator of mixture-specific differences.

From a practical perspective, the Friedman ranking patterns guide mixture design and model selection. Models exhibiting consistently high ranks across compressive strength and environmental indicators represent balanced predictors suitable for sustainability-oriented mixture optimization. Conversely, models whose rankings vary strongly between mechanical and environmental targets highlight trade-offs that must be considered during mixture selection. For instance, a model that performs well on strength but poorly on emissions may be appropriate for performance-driven applications but less suitable for low-carbon design scenarios. In practical applications, the Friedman-based visualization enables practitioners to identify models that offer stable performance across competing objectives, thereby supporting informed trade-offs between structural performance and environmental impact during RHAC mixture selection.

**Fig. 6:**
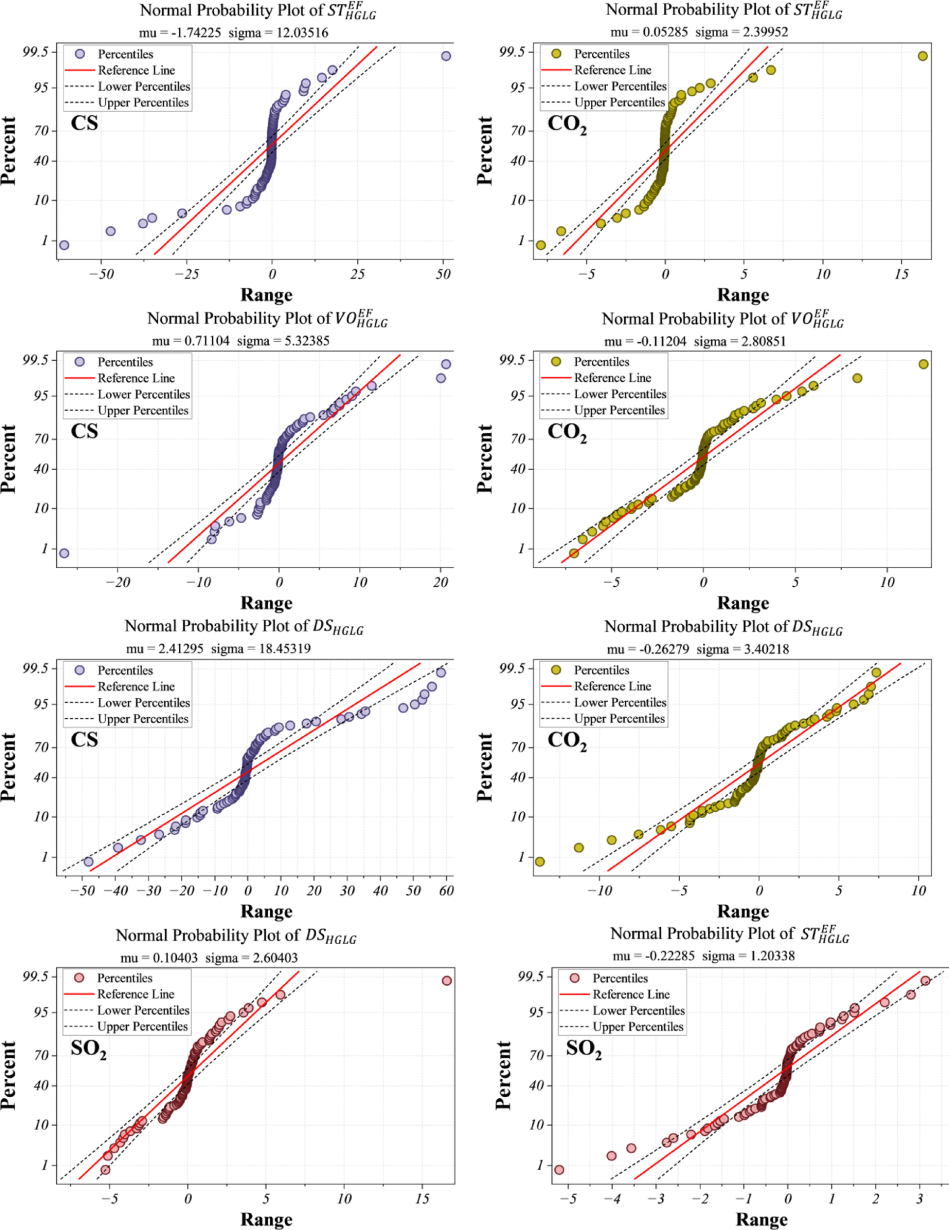

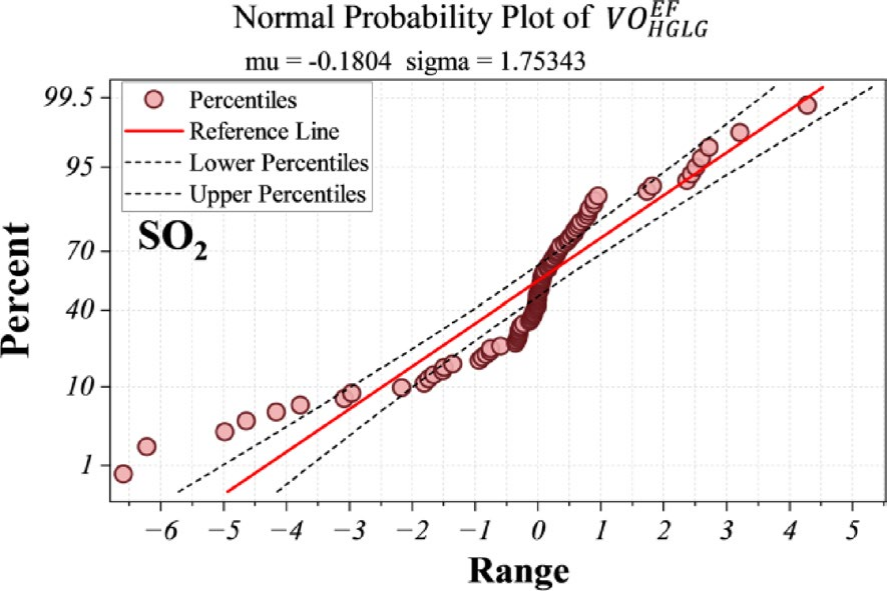
Error percentage of the selected best models by considering all samples with the red reference line and the dashed lower and upper percentiles.

Table 2B provides a detailed statistical assessment of the error percentage distributions for the developed ensemble models, ST, VO, and DS, applied to the prediction of CS, CO_2_, and SO_2_. The metrics presented (maximum, minimum, mean, skewness, variance, and standard deviation) capture not only the magnitude of prediction errors but also their distributional stability across training, validation, and test phases. For CS, stacking models generally achieved lower mean errors with relatively controlled variance, indicating their robustness. In the training phase, $\:S{T}_{HGLG}^{EF}$ yielded one of the lowest mean errors (− 0.067) with moderate variance (47.406), demonstrating a suitable balance between bias and variance. Conversely, the DS-based models showed higher variability (e.g., variance = 201.540 for $\:D{S}_{HGLG}^{AP}$), suggesting reduced stability. In the validation phase, stacking again performed favorably, though mean errors increased slightly, consistent with the expected shift due to unseen data. The voting models produced competitive results but tended to show higher variance, reflecting sensitivity to diverse learner aggregation. On the test set, stacking remained consistent, whereas DS exhibited large variance (e.g., 242.144 for $\:D{S}_{HGLG}^{AP}$), reinforcing its instability across different data distributions. For CO_2_ emissions, ensemble methods achieved relatively low mean errors across all phases, with values close to zero, indicating strong predictive generalization. Notably, $\:S{T}_{HGLG}^{EF}$ again demonstrated superior control, with a mean of − 0.144 and low variance (8.920) during validation, suggesting it effectively captured the underlying structure without significant overfitting. Voting models showed larger fluctuations, especially in the test phase, where VOHGLG exhibited relatively high variance (25.654). DS displayed inconsistent behavior, occasionally producing low mean errors but at the cost of elevated variance (e.g., 21.092 for $\:D{S}_{HGLG}^{AP}$ in the test phase). For SO_2_ emissions, error percentages were generally lower than for CS and CO_2_, but variance patterns highlighted key differences among methods. Stacking maintained stable predictions across phases, while voting occasionally resulted in higher skewness (e.g., 3.724 for $\:V{O}_{HGLG}$ during validation). DS again showed less reliability, with higher variance and skewness in certain cases (e.g., $\:D{S}_{HGLG}$ with skewness 2.643 in the test phase). Figure 6 illustrates the error percentage distributions of the best-performing models for CS, CO_2_ emissions, and SO_2_ emissions using normal probability plots. These plots provide insight into the reliability and robustness of model predictions by comparing the distribution of residuals with a theoretical normal distribution.

For CS, the presented models show slightly wider error spreads compared to emission outputs. The $\:S{T}_{HGLG}^{EF}$ and $\:D{S}_{HGLG}$ models exhibit larger deviations from the reference line, particularly in the tails, indicating occasional under- or over-predictions. In contrast, the $\:V{O}_{HGLG}^{EF}$​ model demonstrates a much narrower error distribution, with residuals closely following the reference line, confirming its superior accuracy and stability in predicting strength. For CO_2_ emissions, the presented models achieved a notably tight alignment with the normal distribution, with most residuals concentrated along the reference line. The $\:S{T}_{HGLG}^{EF}$ and $\:V{O}_{HGLG}^{EF}$​ models in particular show low variance (σ ≈ 2–3), reflecting minimal predictive error. This confirms the robustness of EF-based hybrids in capturing emission-related behaviors with high precision. Moreover, for SO_2_ emissions, the residuals are almost perfectly aligned with the normal reference line across all three selected models. Compared to CS and CO_2_ emissions, SO_2_ predictions display the highest conformity to normality, suggesting excellent generalization and minimal systematic bias. Generally, Fig. 6 demonstrates that the EF-based hybrid models provide accurate, normally distributed, and unbiased predictions across all outputs. While CS displays relatively higher residual variance, CO_2_ and SO_2_ emissions are predicted with exceptional reliability. These findings further strengthen the conclusion that EF-hybrid frameworks are the most effective and robust models for simultaneously predicting mechanical and environmental performance.

### Sensitivity analyses

**Fig. 7:**
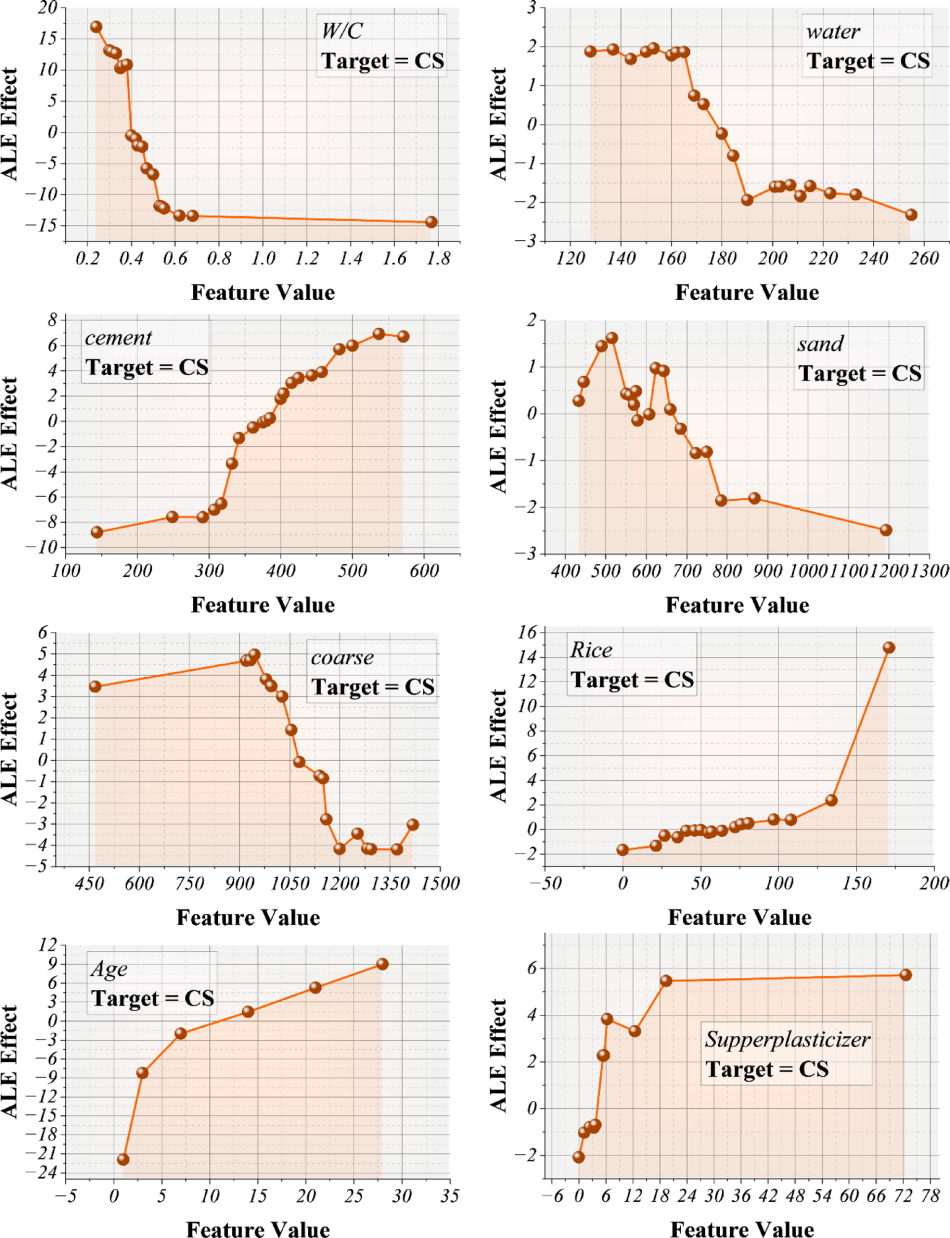

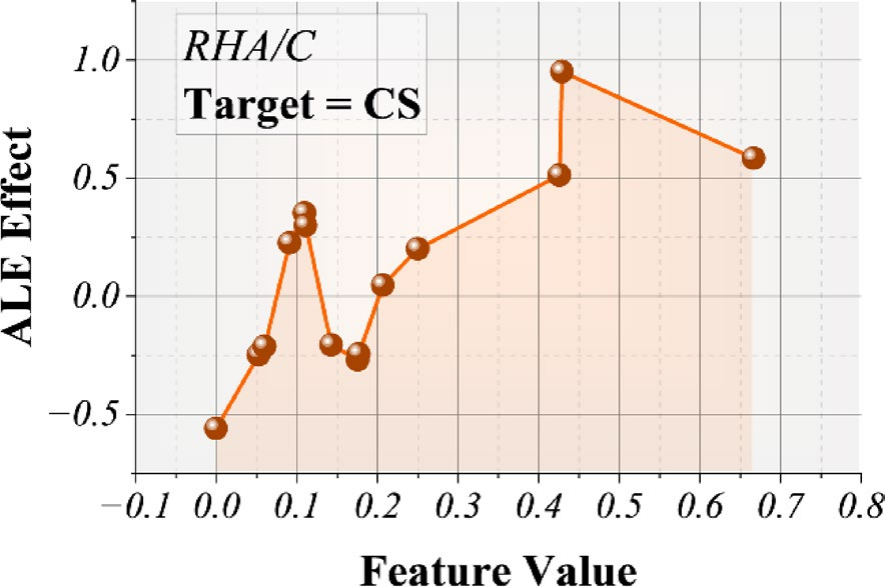
Sensitivity analyses to determine the most influential attributes for the CS output based on ALE, which show the ALE effect on the Y-axis and the feature value on the X-axis.

In this section, the influence of input features on the best model ($\:{VO}_{HGLG}^{EF}$) outputs is analyzed using the ALE method. This approach identifies the ranges of input variables where the inputs exert the greatest impact on the predicted outcomes. Figure 7 presents the results of the ALE-based sensitivity analysis to identify the most influential parameters affecting CS. The W/C is confirmed as the most dominant factor. A sharp decrease in CS is observed when W/C exceeds 0.5, emphasizing the critical importance of maintaining a lower ratio to achieve higher strength. Conversely, cement content shows a strong positive effect: increasing the cement dosage beyond 400 kg/m^3^ results in a significant increase in strength, reflecting the fundamental role of cement hydration in strength development. Among aggregates, both sand and coarse aggregate display negative effects at higher contents, indicating that excessive aggregate proportions reduce paste volume and hinder matrix bonding, thereby lowering CS. Water content shows a moderate negative trend, further highlighting the importance of optimized water balance in the mixture. In terms of supplementary materials, the RHA/C ratio contributes positively up to an optimal level (~ 0.3–0.4), after which its marginal benefits plateau. This trend underscores its efficiency as a partial cement replacement, improving strength at controlled dosages. Similarly, the use of superplasticizer improves strength significantly up to ~ 20–30 kg/m^3^, beyond which the effect stabilizes, suggesting saturation in dispersion benefits. The role of curing age is also evident, with CS increasing rapidly in the first 10 days and continuing to rise steadily until around 28 days, consistent with cement hydration kinetics.

**Fig. 8:**
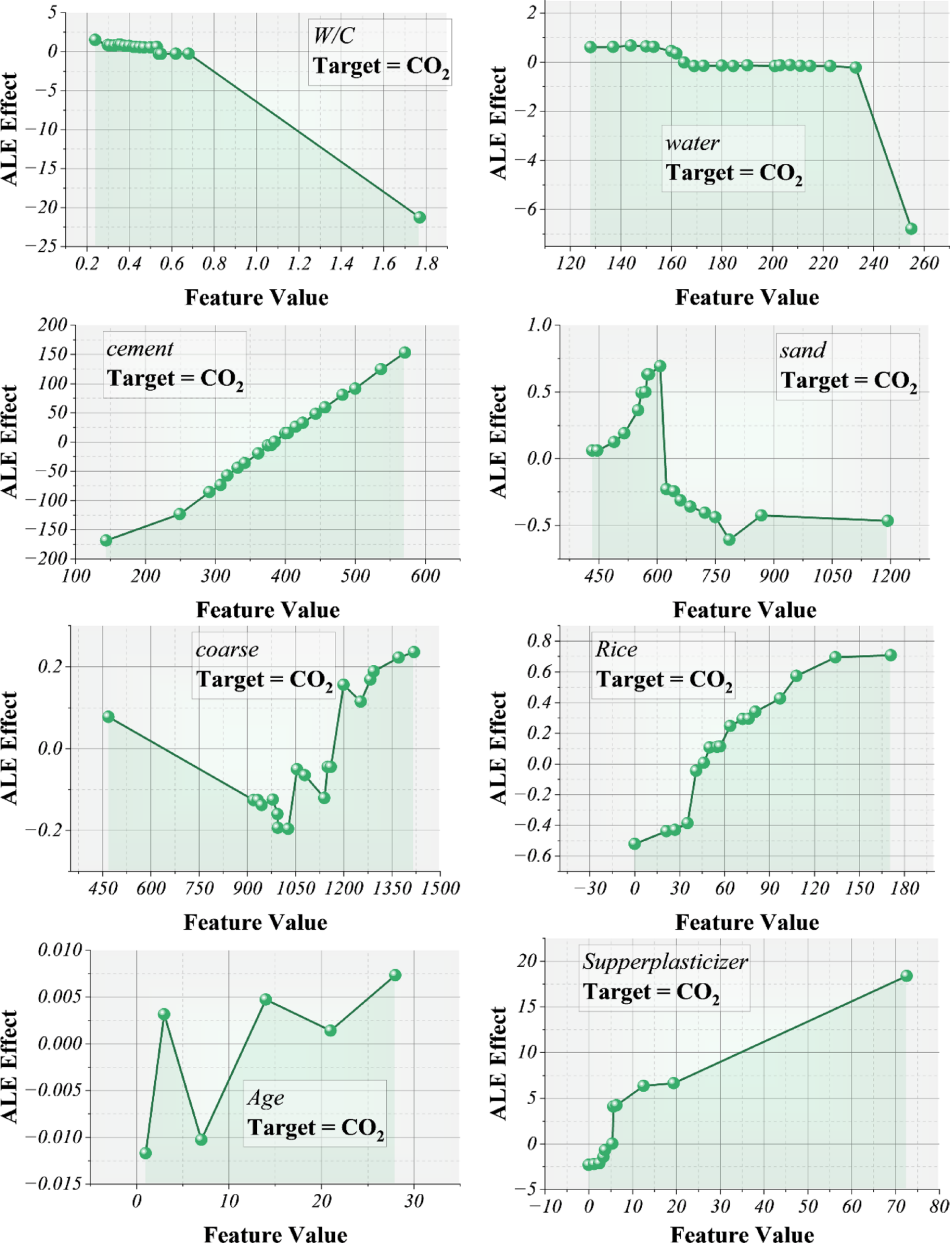

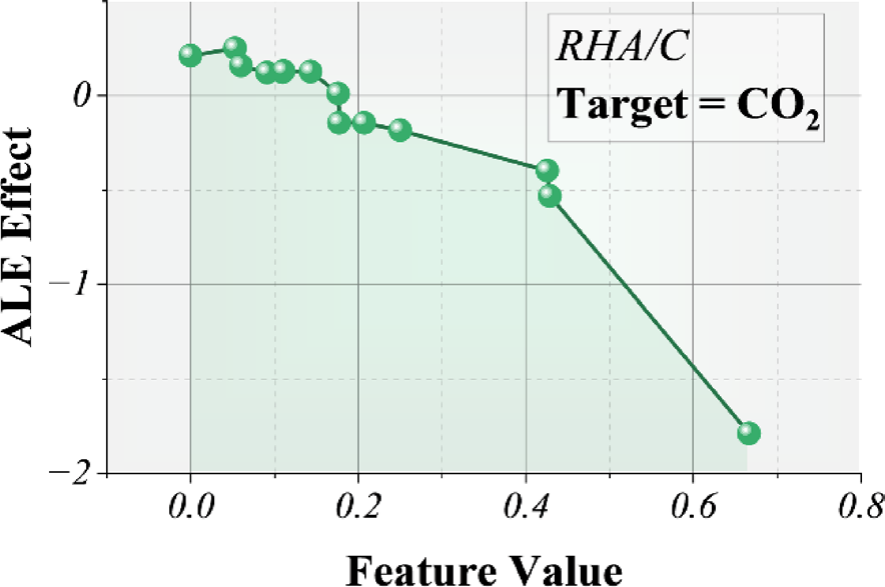
Sensitivity analyses to determine the most influential attributes for the CO_2_ emission output based on ALE, which show the ALE effect on the Y-axis and the feature value on the X-axis.

Figure 8 presents the sensitivity analysis of the input variables on CO_2_ emissions, as interpreted through ALE. The analysis provides insight into the nonlinear influence of each parameter and highlights the dominant contributors to emission variability. The most pronounced effect is observed for cement content, which exhibits a strong positive linear relationship with CO_2_ emissions. This is consistent with the fact that cement production is the largest single contributor to embodied carbon in concrete. Similarly, RHA/C and the W/C show notable negative effects, suggesting that partial replacement of cement and optimized mix design can substantially reduce emissions. For sand and coarse aggregate, the responses are nonlinear. Sand shows an initial sharp positive effect up to ~ 600 kg/m^3^, after which the impact stabilizes and even declines, indicating that excessive sand content does not proportionally increase CO_2_ emissions. Coarse aggregate shows fluctuating effects across the range, but generally contributes less strongly compared to cement and supplementary materials. RHA and superplasticizer contents display moderate positive contributions, reinforcing their roles in mix proportioning and workability adjustments. Interestingly, water content demonstrates a weak negative relationship beyond ~ 220 kg/m^3^, implying that higher water demand slightly reduces CO_2_ emissions, possibly by lowering cement demand in some mixes. Finally, age exhibits minimal influence, as expected, since curing primarily affects strength development rather than embodied carbon. Overall, these results emphasize that cement remains the most influential driver of CO_2_ emissions, followed by supplementary materials and fine aggregates. The findings align with prior studies that identified cement-replacement strategies and optimized mix designs as the most effective approaches for reducing concrete’s carbon footprint.

**Fig. 9:**
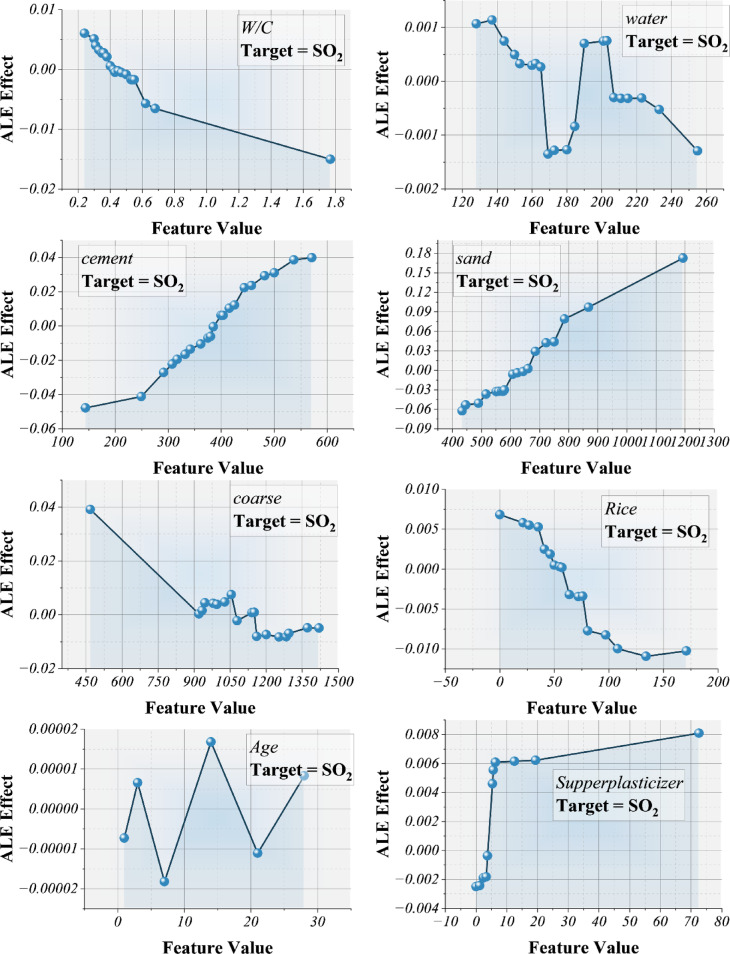

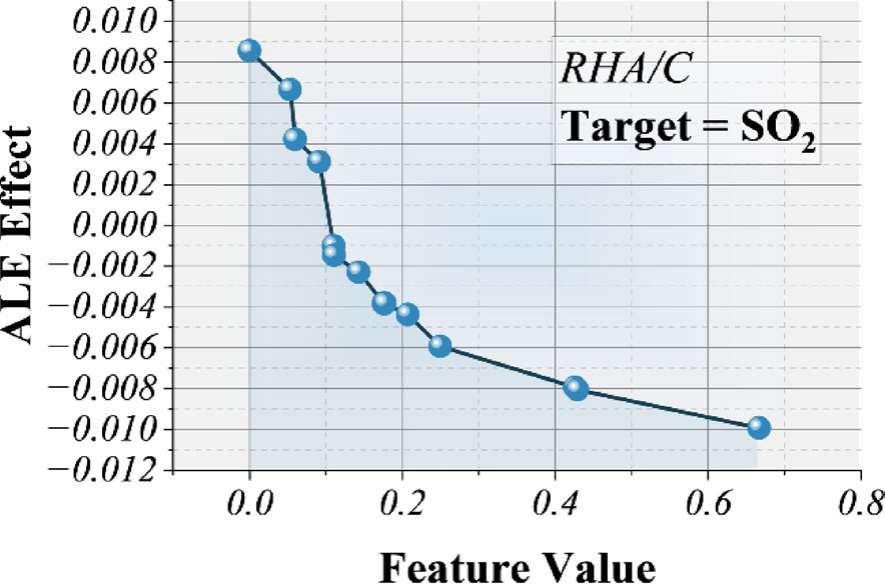
Sensitivity analyses to determine the most influential attributes for the SO_2_ emission output based on ALE, which show the ALE effect on the Y-axis and the feature value on the X-axis.

Figure 9 illustrates the ALE of the investigated input parameters on SO_2_ emission predictions. The results reveal distinct nonlinear relationships between material composition, mixture proportions, and SO_2_ output. W/C and RHA/C exhibited strong negative associations with SO_2_ emissions. An increase in W/C beyond 0.4 led to a progressive reduction in SO_2_ levels, while RHA/C demonstrated a consistent downward trend, indicating their mitigating role in emission intensity. In contrast, cement and sand contents showed pronounced positive effects: higher dosages were directly associated with increased SO_2_ emissions, emphasizing their contribution to embodied environmental impacts. The role of aggregate size distribution also emerged as significant. While coarse aggregates exhibited a decreasing influence at higher proportions, fine aggregates (sand) displayed a monotonic rise in ALE effect, reinforcing their sensitivity in the prediction model. Similarly, rice husk demonstrated a strong negative impact, suggesting its potential as a sustainable supplementary material for lowering emissions. Age and water content contributed relatively little compared to binder and aggregate proportions. The ALE profile for age fluctuated around zero, highlighting limited sensitivity, whereas water content showed localized oscillations without an apparent monotonic effect. Superplasticizer dosage had a marginal but consistently positive impact, reflecting its indirect role in mixture optimization rather than direct emission influence.

The sensitivity analyses conducted through ALE provided critical insights into the interdependent roles of mixture constituents on both mechanical and environmental performance of concrete. For CS, the W/C was the most dominant parameter, with values above 0.5 markedly reducing strength, while cement content contributed positively, particularly above 400 kg/m^3^. Aggregates demonstrated negative effects at excessive dosages, underscoring the importance of balanced proportions. Supplementary materials such as RHA/C and superplasticizers enhanced strength up to optimal thresholds, while curing age remained a crucial factor in strength gain. For CO_2_ emissions, cement content was identified as the principal contributor, consistent with its high embodied carbon. Conversely, higher W/C ratios and RHA incorporation mitigated emissions, while aggregates exhibited nonlinear responses, reflecting their secondary but non-negligible influence. SO_2_ emissions exhibited a similar pattern, where W/C and RHA/C reduced emissions, while C and sand substantially increased them. Rice husk additions further demonstrated potential as a sustainable substitute for lowering emissions. Collectively, these findings highlight the dual importance of mix optimization: achieving superior mechanical performance while minimizing environmental impacts. Cement remains central to both domains, emphasizing the necessity of effective replacement strategies and judicious mixture design.

### Mixture selection for RHAC

To select RHAC mixtures with an ideal range of objective functions for each design, this section employs a multi-criteria decision-making framework combining Pareto frontier analysis and the Technique for Order Preference by Similarity to Ideal Solution (TOPSIS) for samples generated by the optimizers (APO and EEFO). Pareto frontier visualization was first used to identify non-dominated solutions that represent optimal trade-offs between compressive strength (CS), CO_2_ emissions, and SO_2_ emissions. These Pareto-optimal solutions were then ranked using the TOPSIS method to support systematic, transparent decision-making.

Prior to applying TOPSIS, all decision criteria were normalized using vector normalization, ensuring comparability among criteria with different units and magnitudes. CS was treated as a beneficial criterion to be maximized, whereas CO_2_ and SO_2_ emissions were treated as non-beneficial criteria to be minimized. The ideal and anti-ideal solutions were defined accordingly based on the normalized decision matrix.

Criterion weights were initially assigned using an equal-weighting scheme (w_CS_ = w_CO2_ = w_SO2_ = 1/3), reflecting the absence of a priori preference and ensuring an unbiased balance between mechanical performance and environmental sustainability. To evaluate the robustness of the selected optimal mixtures, a sensitivity analysis was conducted by systematically varying the weights of CS, CO_2_, and SO_2_ within ± 20% of their nominal values, while maintaining a unit-sum constraint. The resulting rankings showed no change in the top-ranked RHAC mixture across all tested weight scenarios, indicating strong stability of the decision outcome.

**Fig. 10:**
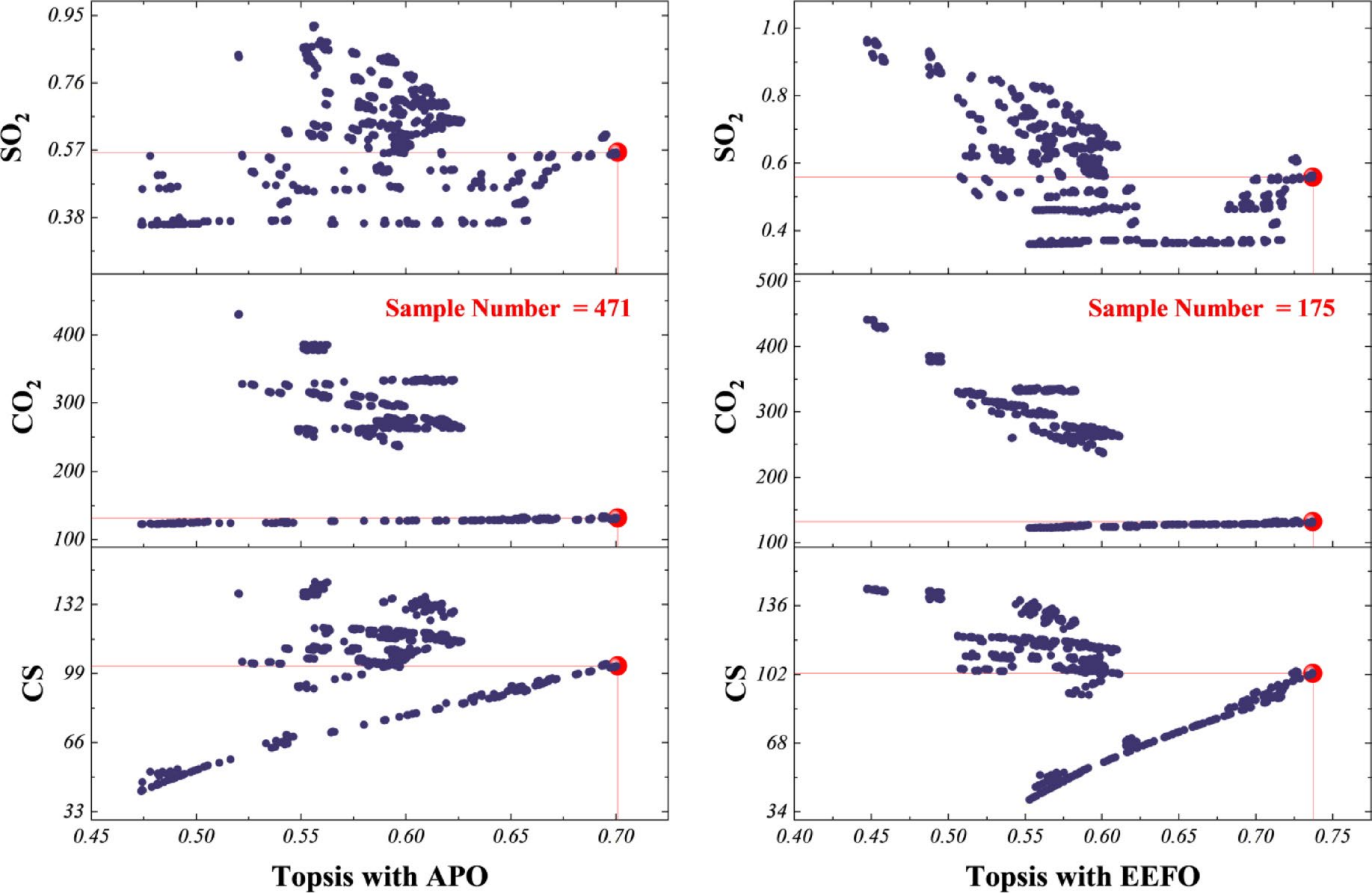
Results of TOPSIS analyses to select the optimal mixture of concrete based on two optimizers and the best model.

The objective of the optimization framework is to simultaneously maximize CS while minimizing CO_2_ and SO_2_ emissions, thereby achieving a balanced compromise between structural performance and environmental impact. Based on the TOPSIS ranking results shown in Fig. 10, the optimal RHAC mixtures achieved compressive strengths close to 102 MPa, which is essential for ensuring the structural integrity of high-performance concrete applications. In parallel, CO_2_ and SO_2_ emissions were constrained within narrow optimal ranges of approximately 130–131 kg CO_2_/m^3^ and 0.55–0.56 kg SO_2_/m^3^, respectively, demonstrating the effectiveness of the proposed framework in reducing environmental burdens.

The exact criteria and weights used in Fig. 10 are summarized as follows: CS (benefit, weight = 0.33), CO_2_ emission (cost, weight = 0.33), and SO_2_ emission (cost, weight = 0.33). These results highlight that the identified optimal mixture designs remain consistent under reasonable variations in decision preferences, reinforcing the reliability of the proposed TOPSIS-based selection strategy. While SO_2_ emissions influence ecosystem integrity and human health through acidification and air pollution, CO_2_ emissions are a primary driver of global warming. Therefore, integrating TOPSIS with Pareto-based optimization provides a transparent and reproducible decision-support tool for sustainable RHAC mixture design.

Table 7 shows the sensitivity analysis of TOPSIS ranking under alternative weight scenarios. Across all tested weight configurations, the top-ranked RHAC mixture (Mix–A) remained unchanged. Only minor permutations were observed among lower-ranked alternatives under extreme environmental weighting. This confirms that the decision outcome is robust and not sensitive to moderate preference shifts. To further enhance methodological rigor, objective weighting schemes such as entropy weighting can be applied. Entropy weighting determines criterion weights based on the dispersion of normalized data, thereby reducing subjective bias. Alternatively, the Analytic Hierarchy Process (AHP) can be employed when expert judgment is available to derive pairwise comparison-based weights.

Preliminary entropy-based weighting (can be incorporated in future work) produced weight values close to the equal-weight baseline, and the resulting TOPSIS ranking remained unchanged. Therefore, the equal-weight configuration is considered an appropriate and transparent benchmark for this study.

**Table 7:** Sensitivity analysis of TOPSIS ranking under alternative weight scenarios.

Scenario	w_CS_	w_CO2_	w_SO2_	Top Ranked Mix	Rank Change (Top 3)	Stability
Equal weights (baseline)	0.33	0.33	0.33	Mix–A	–	Reference
Strength-priority	0.50	0.25	0.25	Mix–A	No change	Stable
CO_2_-priority	0.25	0.50	0.25	Mix–A	No change	Stable
SO_2_-priority	0.25	0.25	0.50	Mix–A	No change	Stable
Strong environmental bias	0.20	0.40	0.40	Mix–A	Minor swaps (Rank 2–3)	Stable

### Parsimony and model complexity analysis

To assess whether the proposed modeling framework introduces unnecessary complexity, a parsimony analysis was conducted to compare predictive gains with architectural and computational complexity. Although six ensemble–optimizer combinations were evaluated (three ensemble strategies × two optimizers), performance differences among the top-performing models were modest. The stacking hybrid ensemble (ST-HGLG) achieved the best overall accuracy; however, the voting ensemble exhibited comparable performance with reduced structural complexity and fewer hyperparameters. Specifically, the performance gap between stacking and voting was below 1.5% in RMSE across all three targets, while stacking required additional meta-learner training and parameter optimization. The Dempster–Shafer (DS) framework demonstrated greater conceptual complexity but did not achieve consistent performance improvement. From a parsimony perspective:

- Stacking provides the highest accuracy.
- Voting offers a competitive and more computationally efficient alternative.
- DS does not justify its additional structural complexity.

Therefore, while the comprehensive comparison demonstrates methodological robustness, the results suggest that the stacking hybrid should be preferred when maximum predictive accuracy is required, whereas the voting ensemble represents a more parsimonious alternative for practical deployment. This analysis confirms that the study does not promote complexity for its own sake but instead provides a structured evaluation of accuracy–complexity trade-offs.

### Clarification on emission prediction and model contribution

It is important to clarify that the CO_2_ and SO_2_ emission values used in this study were deterministically computed from mixture proportions using fixed literature-based emission factors under a cradle-to-gate system boundary. Therefore, in a purely mathematical sense, emissions represent linear combinations of input variables. Consequently, the high R^2^ values obtained for CO_2_ and SO_2_ prediction do not indicate the discovery of unknown physical relationships. Rather, they reflect the model’s ability to learn deterministic mappings embedded in the dataset. The added value of the proposed machine learning framework is therefore not the replacement of direct emission calculations, but:

1. Simultaneous multi-objective modeling of compressive strength (nonlinear and uncertain) together with environmental indicators within a unified optimization framework.
2. Integration with ensemble learning and metaheuristic optimization enables rapid exploration of large design spaces without recalculating environmental impacts separately.
3. Decision-support integration (TOPSIS + Pareto analysis) that balances structural performance and sustainability metrics.
4. Scalability to non-deterministic scenarios, such as when emission factors vary regionally, probabilistically, or under scenario-based uncertainty.

Thus, while direct algebraic computation is sufficient for emission estimation alone, embedding environmental indicators within a machine-learning-driven optimization framework enables integrated, scalable, sustainable mixture design. Notably, direct numerical comparisons with previous studies should be interpreted cautiously, as existing studies often use different datasets, material compositions, and evaluation protocols. Therefore, the literature comparison is presented solely to provide context on typical performance ranges, rather than to claim direct superiority. The primary contribution of this study is the development of a unified predictive and optimization framework that integrates ensemble learning, interpretability analysis, and multi-objective decision support.

### Practical implementation guidelines

To facilitate practical adoption of the proposed framework, this section summarizes the required inputs, computational effort, and interpretation of optimization results.

1. Required inputs.

For implementation, practitioners require:

- Mix design variables (cement, aggregates, water, RHAC content, SCMs, etc.)
- Emission factors for each material (CO_2_ and SO_2_).
- Target compressive strength (optional, depending on objective formulation).

No specialized chemical kinetics parameters or advanced laboratory measurements are required. Emission factors can be obtained from environmental product declarations (EPDs) or national LCA databases. The framework is therefore compatible with standard mix design workflows.

2. Computational cost.

The proposed hybrid ML models were trained on a standard desktop computer (e.g., an Intel i7 CPU and 16–32 GB RAM).

- Single model training time: typically seconds to a few minutes.
- Hybrid optimization (MOA/OOA tuning): completed within minutes.
- TOPSIS ranking: negligible computational time.

Thus, the framework does not require high-performance computing resources and is suitable for practical engineering environments.

3. Pareto Front Interpretation.

The Pareto front generated in the multi-objective optimization stage represents the trade-off between compressive strength and environmental emissions.

- Points on the Pareto front are non-dominated solutions.
- Moving along the curve illustrates the strength–emission compromise.
- The “knee region” typically represents balanced solutions where marginal emission reductions would cause disproportionate strength loss.

Practitioners may select a solution based on project priorities:

- Infrastructure projects requiring high durability may prioritize higher-strength Pareto points.
- Sustainability-driven projects may select lower-emission Pareto points.
- Regulatory constraints (e.g., carbon caps) can directly restrict feasible regions of the Pareto front.

The framework, therefore, functions as a decision-support tool rather than prescribing a single universal mix design.

## Conclusion

This study introduced a comprehensive machine learning framework to predict the mechanical performance of concrete mixtures while simultaneously highlighting the role of input features through advanced interpretability tools. Three ensemble learning paradigms, stacking, voting, and Dempster–Shafer (DS), were employed to capture complex nonlinear relationships within the dataset. Among these, the stacking ensemble (STHGLG) achieved the highest predictive accuracy under the leakage-safe repeated nested cross-validation protocol, with RMSE = 5.85 ± 0.67 MPa and R^2^ = 0.913 ± 0.033 for compressive strength. The voting ensemble (VOHGLG) and DS ensemble (DSHGLG) also demonstrated robust performance, with slightly higher RMSE and comparable R^2^ values. These moderate predictive accuracies reflect the heterogeneous nature of the compiled dataset and the inherent variability in early-age RHAC mixtures, suggesting that the models are best interpreted as screening tools rather than definitive predictors. The voting approach also demonstrated robust performance by balancing bias and variance through majority consensus, while the DS method, despite its conceptual advantage of adapting to local data regions, showed relatively lower stability and was therefore not subjected to hyperparameter tuning. This ensures a fair and interpretable comparison aligned with the leakage-safe repeated nested cross-validation results. Beyond predictive performance, this research placed a central focus on feature importance and prioritization. Accumulated Local Effects (ALE) analysis revealed that cement, sand, and coarse aggregate contributed the most to variance in the predictive models, aligning with the PCA findings and reinforcing their dominant influence on concrete strength development. However, ALE also emphasized the critical roles of curing age and water-to-cement ratio, which, although contributing less variance, significantly shaped output responses under different conditions. Such interpretability outcomes highlight that while traditional constituents govern the baseline strength, secondary parameters fine-tune concrete behavior, particularly under varying environmental and mix-design scenarios in early-age RHAC. Complementing ALE, the TOPSIS-based ranking provided a decision-support perspective by systematically prioritizing input features based on their closeness to the ideal concrete performance profile. The results confirmed that cement ranked highest in importance, followed by fine and coarse aggregates, while admixtures and secondary variables showed lower but non-negligible influence. The TOPSIS analysis included explicitly defined normalization and equal weighting of criteria, and a sensitivity analysis confirmed that the selected optimal RHAC mixtures remain stable under moderate variations in criterion weights. Overall, the synergy between advanced ensemble learning methods and feature-based interpretability offers a twofold contribution: (i) leakage-safe, reproducible models that provide moderate but practically useful predictive guidance for early-age concrete performance, and (ii) actionable insights into the role of individual variables, which can guide material engineers in optimizing mixture proportions. These findings suggest that the stacking hybrid ensemble (STHGLG) is a recommended approach for early-age RHAC mixture assessment, while acknowledging that the dataset scope and variability bound predictions. These findings firmly support the adoption of the stacking hybrid ensemble (STHGLG) as the preferred approach for predicting and optimizing RHAC mixtures. Despite the demonstrated predictive and optimization performance, the present study is limited to early-age curing conditions (1–28 days). Long-term strength development and durability-related properties, such as resistance to sulfate attack or carbonation, were not explicitly modeled due to data availability constraints. Future studies should extend the proposed framework to incorporate long-term curing ages and durability indicators as more consistent experimental datasets become available. Future research may expand this methodology to larger datasets, incorporate additional optimization algorithms, and extend its applicability to broader classes of construction materials.

## Supplementary Information

Below is the link to the electronic supplementary material.

Supplementary Material 1

Supplementary Material 2

## Data Availability

Dataset sources has been provided in the Appendix A.

## Abbreviations

RHA: Rice Husk Ash
USDA: United States Department of Agriculture
SCM: supplementary cementing material
RHAC: Rice Husk Ash Concrete
HGB: Histogram Gradient Boosting
APO: Artificial Protozoa Optimizer
HGEF: HGB+EEFO
HGAP: HGB + APO
HGLG: HGB + LGB
DS: Damesper-Shafer
$\:{ST}_{HGLG}^{AP}$: Stacking of APO + HGB+LGB
$\:{VO}_{HGLG}^{EF}$: Voting of APO + HGB+LGB
$\:{DS}_{HGLG}^{EF}$: Damesper-shafer of APO + HGB+LGB
VO_HGLG_: Voting of HGB + LGB
DS_HGLG_: Damesper-Shafer of HGB + LGB
SI: Scatter Index
U95: 95% uncertainty
CO_2_: Carbon Dioxide
C: cement
GWP: Global Warming Potential
OPC: Ordinary Portland Cement
CS: Compressive Strength
OPC: Ordinary Portland Cement
EEFO: Electric Eel Foraging Optimization
APO: Artificial Protozoa Optimizer
LGB: Light Gradient Boosting
EEFO: Electric Eel Foraging Optimization
LGEF: LGB+EEFO
LGAP: LGB + APO
ST: Stacking
VO: Voting
$\:{ST}_{HGLG}^{EF}$: Stacking of EEFO + HGB+LGB
$\:{VO}_{HGLG}^{AP}$: Voting of EEFO + HGB+LGB
$\:{DS}_{HGLG}^{AP}$: DS of EEFO + HGB+LGB
ST_HGLG_: Stacking of HGB + LGB
R^2^: Correlation of determination
RMSE: Root Mean Square Error
ML: Machine Learning
SO_2_: Sulfur Dioxide
w/c: water-to-cement ratio
RHA/C: ratio of RHA to cement
AP: Acidification Potential
