# Supplementary material for: Sustainable mixture design of rice husk ash cement based concrete: performance optimization through data driven modeling and multi objective analysis
Source: Sci Rep. 2026 Apr 19;16:18108. doi: 10.1038/s41598-026-47921-9 (PMC13254211; doi:10.1038/s41598-026-47921-9)
Supplement: Supplementary file 1 — Supplementary Material 1 [file 41598_2026_47921_MOESM1_ESM.docx]

**Appendix A:** A summarizes the primary literature sources from which the RHAC dataset was compiled. For each study, the number of collected samples, mixture composition ranges (water, cement, fine aggregate, coarse aggregate, RHA, and superplasticizer), curing ages, and measured CS values are reported. This compilation highlights the diversity of mixture proportions and testing conditions across different studies, which collectively provide a comprehensive basis for the present analysis.

| **Table 1A.** Sources of RHAC data and corresponding ranges of mixture compositions, curing ages, and CS values. | | | | | | | | | |
| --- | --- | --- | --- | --- | --- | --- | --- | --- | --- |
| **Ref.** | **Num. of data** | **Water (kg/m³)** | **Cement (kg/m³)** | **F.A. (kg/m³)** | **C.A. (kg/m³)** | **RHA (kg/m³)** | **Age (days)** | **SP (kg/m³)** | **CS (MPa)** |
| [39] | 27 | 164–204 | 327–534 | 690–758 | 983–1050 | 0–85 | 7, 28, 91 | 0.462–4.33 | 29.22–77.96 |
| [40] | 20 | 150 | 375 | 770 | 1200 | 0–75 | 7, 28, 56, 90 | 0–1.5 | 30–64 |
| [41] | 16 | 138–173 | 322–513 | 720–845 | 975–1045 | 0–34 | 28, 90 | 0–10,26 | 37–82.1 |
| [42] | 20 | 150–153 | 392–560 | 735–764 | 943–981 | 0–168 | 7, 28, 60, 90, 180 | 1.5–9.0 | 103.06–118.82 |
| [43] | 21 | 201 | 266–380 | 570 | 1140 | 0–114 | 7, 14, 28 | 0 | 27.22–39.55 |
| [44] | 32 | 203 | 249–383 | 561 | 1148 | 0–134 | 7, 14, 28, 90 | 0 | 27.22–45.98 |
| [45] | 32 | 131.97–202.99 | 249–383 | 575 | 1150 | 0–134 | 1, 3, 7, 28 | 0 | 10.4–46.7 |
| [46] | 60 | 137–190 | 304–500 | 745–868 | 933–995 | 0–100 | 1, 3, 7, 28 | 0–0.388 | 19–68.6 |
| [47] | 12 | 207 | 313–391 | 750 | 994 | 0–78 | 1, 3, 7, 28, 90, 180 | 0 | 19.1–48.1 |
| [48] | 16 | 157–173 | 492–518 | 484–510 | 983–1025 | 0–52 | 7, 28, 90, 180 | 0 | 66.3–93.5 |
| [49] | 48 | 215–255 | 300–351 | 580–710 | 1160–1185 | 0–134 | 7, 14, 28, 90 | 0 | 13.27–47.6 |
| [50] | 20 | 207 | 313–391 | 750 | 994 | 0–78 | 1, 3, 7, 28 | 0 | 17.2–50.2 |
| [51] | 24 | 122.4–150.5 | 340–430 | 332–335 | 1012–1014 | 0–64.5 | 7, 28, 90 | 0 | 30.36–62.5 |
| [52] | 5 | 192 | 278.7–348.4 | 573 | 1189.5 | 0–69.6 | 28 | 0 | 16.03–29.3 |
| [53] | 10 | 125–156 | 312–390 | 713 | 1079 | 0–78 | 7, 28 | 1.85–3.51 | 30.22–48.53 |
| [54] | 5 | 130 | 418.5–465 | 556–562 | 1268.3–1280.9 | 0–46.5 | 14 | 0 | 46.2–52.6 |
| [55] | 16 | 138–207 | 400–571 | 578–612 | 1027–1088 | 0–171 | 3, 7, 28, 150 | 1.0–1.4275 | 31.5–85 |
| [56] | 27 | 185 | 261.37–461.25 | 582–623.79 | 1204–1287.9 | 0–69.19 | 7, 14, 28 | 0 | 19.76–43.16 |
| [57] | 8 | 153 | 238–340 | 763 | 1144 | 0–102 | 28 | 3.4–9.2 | 28.38–34.98 |
| [58] | 35 | 140–167 | 456–537 | 516–669 | 1055 | 0–80.6 | 1, 3, 7, 28, 128 | 5.1–5.37 | 25–103.5 |
| [59] | 12 | 153 | 325.1–382.5 | 482 | 1394.1 | 0–57.37 | 7, 28 | 3.25–3.82 | 25.9–42.45 |
| [60] | 15 | 138 | 240–300 | 660 | 1290 | 0–60 | 7, 14, 28 | 2.4 | 19.86–31.88 |
| [61] | 14 | 153–154 | 345–385 | 667–674 | 1086–1102 | 0–38 | 1, 3, 7, 28, 90, 180, 365 | 2.5–3.9 | 25.34–60.74 |
| [62] | 24 | 128 | 356–410 | 786 | 1044–1062 | 0–72 | 3, 7, 28, 365 | 0 | 42–92 |
| [63] | 18 | 210 | 280–350 | 844–870 | 854–881 | 0–70 | 1, 3, 7, 28, 90, 180 | 0 | 2.4–39.4 |
| [64] | 28 | 178–180 | 356–600 | 570–636 | 906–968 | 0–153 | 3, 7, 28, 90 | 0.89–1.248 | 36.86–106.82 |
| [65] | 60 | 160–170 | 400–550 | 540–567 | 1261–1324 | 0–110 | 1, 3, 7, 28, 90 | 5–6.22 | 18.9–86.8 |
| [66] | 36 | 205–228 | 228–325 | 890–900 | 927–940 | 0–97 | 1, 3, 7, 28, 90, 180 | 0–3.67 | 9.2–45.8 |

**Appendix B:** The performance of the developed models has been presented by evaluation metrics and error percentage in CS, CO_2_ emission, and SO_2_ emission, considering the training, validation, testing, and all samples.

| **Table 1B.** Result of the developed models for the three outputs in specific phases. | | | | | | | | | |
| --- | --- | --- | --- | --- | --- | --- | --- | --- | --- |
| ***Target*** | ***Process*** | **Framework** | Models | ***Evaluation Metrics*** | | | | | |
|  |  |  |  | RMSE | | R^2^ | U95 | SI | NSE |
| **Compressive strength** | ***Training*** | **Ensemble- Hybrid** | ${ST}_{HGLG}^{AP}$ | 3.4792 | | 0.9801 | 9.6376 | 0.0740 | 0.8827 |
|  |  |  | ${ST}_{HGLG}^{EF}$ | 2.3698 | | 0.9906 | 6.5686 | 0.0504 | 0.9304 |
|  |  |  | ${VO}_{HGLG}^{AP}$ | 4.3288 | | 0.9692 | 11.9952 | 0.0920 | 0.8117 |
|  |  |  | ${VO}_{HGLG}^{EF}$ | 3.3347 | | 0.9817 | 9.2430 | 0.0709 | 0.9113 |
|  |  |  | ${DS}_{HGLG}^{AP}$ | 4.7776 | | 0.9629 | 13.2425 | 0.1016 | 0.8049 |
|  |  |  | ${DS}_{HGLG}^{EF}$ | 4.0285 | | 0.9734 | 11.1648 | 0.0857 | 0.8458 |
|  |  | **Ensemble** | ST_HGLG_ | 5.6467 | | 0.9492 | 15.6466 | 0.1201 | 0.7258 |
|  |  |  | VO_HGLG_ | 4.9758 | | 0.9597 | 13.7875 | 0.1058 | 0.7694 |
|  |  |  | DS_HGLG_ | 4.2994 | | 0.9696 | 11.9039 | 0.0914 | 0.8172 |
|  | ***Validation*** | **Ensemble- Hybrid** | ${ST}_{HGLG}^{AP}$ | 3.4682 | | 0.9797 | 9.5038 | 0.0840 | 0.8696 |
|  |  |  | ${ST}_{HGLG}^{EF}$ | 2.6081 | | 0.9882 | 7.1855 | 0.0631 | 0.9348 |
|  |  |  | ${VO}_{HGLG}^{AP}$ | 4.1224 | | 0.9718 | 11.2765 | 0.0998 | 0.7826 |
|  |  |  | ${VO}_{HGLG}^{EF}$ | 4.8009 | | 0.9629 | 13.1240 | 0.1162 | 0.8696 |
|  |  |  | ${DS}_{HGLG}^{AP}$ | 5.6330 | | 0.9505 | 15.3497 | 0.1364 | 0.7283 |
|  |  |  | ${DS}_{HGLG}^{EF}$ | 4.6326 | | 0.9638 | 12.7233 | 0.1121 | 0.8043 |
|  |  | **Ensemble** | ST_HGLG_ | 6.5511 | | 0.9281 | 18.0326 | 0.1586 | 0.7391 |
|  |  |  | VO_HGLG_ | 5.9115 | | 0.9435 | 16.2126 | 0.1431 | 0.7500 |
|  |  |  | DS_HGLG_ | 4.3658 | | 0.9669 | 12.0286 | 0.1057 | 0.8043 |
|  | ***Testing*** | **Ensemble- Hybrid** | ${ST}_{HGLG}^{AP}$ | 3.2153 | | 0.9716 | 8.9110 | 0.0772 | 0.8901 |
|  |  |  | ${ST}_{HGLG}^{EF}$ | 3.9794 | | 0.9589 | 10.9897 | 0.0955 | 0.8901 |
|  |  |  | ${VO}_{HGLG}^{AP}$ | 3.7654 | | 0.9613 | 10.4337 | 0.0904 | 0.8242 |
|  |  |  | ${VO}_{HGLG}^{EF}$ | 1.9325 | | 0.9898 | 5.3359 | 0.0464 | 0.9560 |
|  |  |  | ${DS}_{HGLG}^{AP}$ | 4.3814 | | 0.9498 | 12.0916 | 0.1051 | 0.7912 |
|  |  |  | ${DS}_{HGLG}^{EF}$ | 3.7289 | | 0.9613 | 10.3355 | 0.0895 | 0.8571 |
|  |  | **Ensemble** | ST_HGLG_ | 8.1624 | | 0.8483 | 22.5999 | 0.1959 | 0.7143 |
|  |  |  | VO_HGLG_ | 5.8482 | | 0.9133 | 16.1732 | 0.1403 | 0.7473 |
|  |  |  | DS_HGLG_ | 6.1753 | | 0.9036 | 17.0499 | 0.1482 | 0.7253 |
|  | ***Total*** | **Ensemble- Hybrid** | ${ST}_{HGLG}^{AP}$ | 3.4528 | | 0.9796 | 9.5619 | 0.0752 | 0.8821 |
|  |  |  | ${ST}_{HGLG}^{EF}$ | 2.5981 | | 0.9882 | 7.2015 | 0.0566 | 0.9269 |
|  |  |  | ${VO}_{HGLG}^{AP}$ | 4.2556 | | 0.9691 | 11.7874 | 0.0927 | 0.8100 |
|  |  |  | ${VO}_{HGLG}^{EF}$ | 3.4036 | | 0.9803 | 9.4288 | 0.0741 | 0.9116 |
|  |  |  | ${DS}_{HGLG}^{AP}$ | 4.8332 | | 0.9607 | 13.3879 | 0.1052 | 0.7959 |
|  |  |  | ${DS}_{HGLG}^{EF}$ | 4.0649 | | 0.9718 | 11.2629 | 0.0885 | 0.8428 |
|  |  | **Ensemble** | ST_HGLG_ | 6.0369 | | 0.9401 | 16.7210 | 0.1314 | 0.7260 |
|  |  |  | VO_HGLG_ | 5.1691 | | 0.9551 | 14.3140 | 0.1126 | 0.7653 |
|  |  |  | DS_HGLG_ | 4.5271 | | 0.9651 | 12.5269 | 0.0986 | 0.8068 |
| **Total Co_2_ emission of mixtures** | ***Training*** | **Ensemble- Hybrid** | ${ST}_{HGLG}^{AP}$ | 9.4325 | 0.9842 | | 26.1432 | 0.0285 | 0.9768 |
|  |  |  | ${ST}_{HGLG}^{EF}$ | 6.3988 | 0.9925 | | 17.7291 | 0.0193 | 0.9891 |
|  |  |  | ${VO}_{HGLG}^{AP}$ | 11.5083 | 0.9770 | | 31.8948 | 0.0347 | 0.9659 |
|  |  |  | ${VO}_{HGLG}^{EF}$ | 9.3381 | 0.9842 | | 25.8838 | 0.0282 | 0.9809 |
|  |  |  | ${DS}_{HGLG}^{AP}$ | 13.7595 | 0.9665 | | 38.1382 | 0.0415 | 0.9482 |
|  |  |  | ${DS}_{HGLG}^{EF}$ | 10.5226 | 0.9803 | | 29.1459 | 0.0317 | 0.9754 |
|  |  | **Ensemble** | ST_HGLG_ | 15.6512 | 0.9564 | | 43.3657 | 0.0472 | 0.9263 |
|  |  |  | VO_HGLG_ | 14.2699 | 0.9647 | | 39.5542 | 0.0430 | 0.9359 |
|  |  |  | DS_HGLG_ | 11.1083 | 0.9779 | | 30.7737 | 0.0335 | 0.9686 |
|  | ***Validation*** | **Ensemble- Hybrid** | ${ST}_{HGLG}^{AP}$ | 9.8053 | 0.9767 | | 27.0653 | 0.0302 | 0.9783 |
|  |  |  | ${ST}_{HGLG}^{EF}$ | 7.0579 | 0.9882 | | 19.5616 | 0.0218 | 0.9891 |
|  |  |  | ${VO}_{HGLG}^{AP}$ | 14.4112 | 0.9556 | | 39.9391 | 0.0444 | 0.9130 |
|  |  |  | ${VO}_{HGLG}^{EF}$ | 9.0424 | 0.9814 | | 24.6051 | 0.0279 | 0.9783 |
|  |  |  | ${DS}_{HGLG}^{AP}$ | 14.2903 | 0.9567 | | 39.5448 | 0.0441 | 0.9348 |
|  |  |  | ${DS}_{HGLG}^{EF}$ | 15.4390 | 0.9494 | | 42.5338 | 0.0476 | 0.9565 |
|  |  | **Ensemble** | ST_HGLG_ | 16.8438 | 0.9365 | | 46.5080 | 0.0519 | 0.9022 |
|  |  |  | VO_HGLG_ | 14.7195 | 0.9511 | | 40.6080 | 0.0454 | 0.9565 |
|  |  |  | DS_HGLG_ | 16.8430 | 0.9310 | | 46.6007 | 0.0519 | 0.9130 |
|  | ***Testing*** | **Ensemble- Hybrid** | ${ST}_{HGLG}^{AP}$ | 9.8748 | 0.9836 | | 27.3703 | 0.0300 | 0.9780 |
|  |  |  | ${ST}_{HGLG}^{EF}$ | 6.8810 | 0.9917 | | 19.0726 | 0.0209 | 0.9890 |
|  |  |  | ${VO}_{HGLG}^{AP}$ | 13.0417 | 0.9702 | | 36.1408 | 0.0396 | 0.9341 |
|  |  |  | ${VO}_{HGLG}^{EF}$ | 8.5406 | 0.9873 | | 23.6592 | 0.0260 | 0.9890 |
|  |  |  | ${DS}_{HGLG}^{AP}$ | 13.8336 | 0.9677 | | 38.2926 | 0.0420 | 0.9451 |
|  |  |  | ${DS}_{HGLG}^{EF}$ | 11.4462 | 0.9774 | | 31.6759 | 0.0348 | 0.9780 |
|  |  | **Ensemble** | ST_HGLG_ | 15.7644 | 0.9582 | | 43.6911 | 0.0479 | 0.9341 |
|  |  |  | VO_HGLG_ | 13.3337 | 0.9705 | | 36.8279 | 0.0405 | 0.9670 |
|  |  |  | DS_HGLG_ | 10.4027 | 0.9816 | | 28.7953 | 0.0316 | 0.9670 |
|  | ***Total*** | **Ensemble- Hybrid** | ${ST}_{HGLG}^{AP}$ | 9.5153 | 0.9835 | | 26.3692 | 0.0288 | 0.9771 |
|  |  |  | ${ST}_{HGLG}^{EF}$ | 6.5171 | 0.9921 | | 18.0599 | 0.0197 | 0.9891 |
|  |  |  | ${VO}_{HGLG}^{AP}$ | 11.9891 | 0.9744 | | 33.2293 | 0.0363 | 0.9574 |
|  |  |  | ${VO}_{HGLG}^{EF}$ | 9.2324 | 0.9842 | | 25.5874 | 0.0279 | 0.9814 |
|  |  |  | ${DS}_{HGLG}^{AP}$ | 13.8211 | 0.9656 | | 38.3092 | 0.0418 | 0.9465 |
|  |  |  | ${DS}_{HGLG}^{EF}$ | 11.2054 | 0.9773 | | 31.0371 | 0.0339 | 0.9738 |
|  |  | **Ensemble** | ST_HGLG_ | 15.7862 | 0.9549 | | 43.7329 | 0.0478 | 0.9247 |
|  |  |  | VO_HGLG_ | 14.2258 | 0.9642 | | 39.4318 | 0.0430 | 0.9410 |
|  |  |  | DS_HGLG_ | 11.7467 | 0.9747 | | 32.5464 | 0.0355 | 0.9629 |
| **Total So2 emission of mixtures** | ***Training*** | **Ensemble- Hybrid** | ${ST}_{HGLG}^{AP}$ | 0.0097 | 0.9854 | | 0.0270 | 0.0125 | 1 |
|  |  |  | ${ST}_{HGLG}^{EF}$ | 0.0069 | 0.9928 | | 0.0190 | 0.0088 | 1 |
|  |  |  | ${VO}_{HGLG}^{AP}$ | 0.0136 | 0.9715 | | 0.0376 | 0.0174 | 0.9986 |
|  |  |  | ${VO}_{HGLG}^{EF}$ | 0.0093 | 0.9865 | | 0.0259 | 0.0120 | 1 |
|  |  |  | ${DS}_{HGLG}^{AP}$ | 0.0151 | 0.9648 | | 0.0419 | 0.0194 | 0.9986 |
|  |  |  | ${DS}_{HGLG}^{EF}$ | 0.0115 | 0.9797 | | 0.0317 | 0.0147 | 1 |
|  |  | **Ensemble** | ST_HGLG_ | 0.0165 | 0.9581 | | 0.0456 | 0.0211 | 0.9973 |
|  |  |  | VO_HGLG_ | 0.0141 | 0.9694 | | 0.0390 | 0.0181 | 0.9973 |
|  |  |  | DS_HGLG_ | 0.0113 | 0.9804 | | 0.0313 | 0.0145 | 0.9986 |
|  | ***Validation*** | **Ensemble- Hybrid** | ${ST}_{HGLG}^{AP}$ | 0.0129 | 0.9784 | | 0.0357 | 0.0164 | 1 |
|  |  |  | ${ST}_{HGLG}^{EF}$ | 0.0112 | 0.9843 | | 0.0312 | 0.0143 | 1 |
|  |  |  | ${VO}_{HGLG}^{AP}$ | 0.0161 | 0.9659 | | 0.0446 | 0.0205 | 1 |
|  |  |  | ${VO}_{HGLG}^{EF}$ | 0.0136 | 0.9762 | | 0.0375 | 0.0173 | 1 |
|  |  |  | ${DS}_{HGLG}^{AP}$ | 0.0193 | 0.9526 | | 0.0534 | 0.0246 | 0.9891 |
|  |  |  | ${DS}_{HGLG}^{EF}$ | 0.0148 | 0.9717 | | 0.0410 | 0.0189 | 1 |
|  |  | **Ensemble** | ST_HGLG_ | 0.0195 | 0.9509 | | 0.0537 | 0.0248 | 1 |
|  |  |  | VO_HGLG_ | 0.0167 | 0.9640 | | 0.0463 | 0.0213 | 0.9891 |
|  |  |  | DS_HGLG_ | 0.0147 | 0.9716 | | 0.0406 | 0.0186 | 1 |
|  | ***Testing*** | **Ensemble- Hybrid** | ${ST}_{HGLG}^{AP}$ | 0.0113 | 0.9810 | | 0.0312 | 0.0148 | 1 |
|  |  |  | ${ST}_{HGLG}^{EF}$ | 0.0094 | 0.9872 | | 0.0257 | 0.0122 | 1 |
|  |  |  | ${VO}_{HGLG}^{AP}$ | 0.0152 | 0.9652 | | 0.0420 | 0.0198 | 1 |
|  |  |  | ${VO}_{HGLG}^{EF}$ | 0.0138 | 0.9717 | | 0.0380 | 0.0180 | 1 |
|  |  |  | ${DS}_{HGLG}^{AP}$ | 0.0165 | 0.9610 | | 0.0458 | 0.0216 | 1 |
|  |  |  | ${DS}_{HGLG}^{EF}$ | 0.0178 | 0.9528 | | 0.0491 | 0.0232 | 1 |
|  |  | **Ensemble** | ST_HGLG_ | 0.0212 | 0.9359 | | 0.0580 | 0.0277 | 1 |
|  |  |  | VO_HGLG_ | 0.0174 | 0.9539 | | 0.0483 | 0.0228 | 1 |
|  |  |  | DS_HGLG_ | 0.0180 | 0.9508 | | 0.0499 | 0.0235 | 0.9890 |
|  | ***Total*** | **Ensemble- Hybrid** | ${ST}_{HGLG}^{AP}$ | 0.0103 | 0.9841 | | 0.0284 | 0.0132 | 1 |
|  |  |  | ${ST}_{HGLG}^{EF}$ | 0.0077 | 0.9911 | | 0.0213 | 0.0099 | 1 |
|  |  |  | ${VO}_{HGLG}^{AP}$ | 0.0140 | 0.9703 | | 0.0388 | 0.0180 | 0.9989 |
|  |  |  | ${VO}_{HGLG}^{EF}$ | 0.0104 | 0.9838 | | 0.0287 | 0.0133 | 1 |
|  |  |  | ${DS}_{HGLG}^{AP}$ | 0.0157 | 0.9625 | | 0.0436 | 0.0202 | 0.9978 |
|  |  |  | ${DS}_{HGLG}^{EF}$ | 0.0126 | 0.9760 | | 0.0349 | 0.0162 | 1 |
|  |  | **Ensemble** | ST_HGLG_ | 0.0173 | 0.9547 | | 0.0480 | 0.0222 | 0.9978 |
|  |  |  | VO_HGLG_ | 0.0147 | 0.9672 | | 0.0408 | 0.0189 | 0.9967 |
|  |  |  | DS_HGLG_ | 0.0125 | 0.9765 | | 0.0346 | 0.0160 | 0.9978 |

| **Table 2B.** Statistical metrics to compare the error percentage of the developed models. | | | | | | | | |
| --- | --- | --- | --- | --- | --- | --- | --- | --- |
| ***Phase*** | ***Target*** | ***Models*** | *Properties* | | | | | |
|  |  |  | *Max* | *Min* | *Mean* | *Skew.* | *Var.* | *St. Dev.* |
| ***Train*** | ***CS*** | ${ST}_{HGLG}^{AP}$ | 55.312 | -106.977 | 0.243 | -2.269 | 96.862 | 9.842 |
|  |  | ${ST}_{HGLG}^{EF}$ | 50.711 | -64.174 | -0.067 | -1.740 | 47.406 | 6.885 |
|  |  | ${VO}_{HGLG}^{AP}$ | 81.379 | -51.920 | 0.325 | 0.663 | 130.599 | 11.428 |
|  |  | ${VO}_{HGLG}^{EF}$ | 56.640 | -93.750 | -0.114 | -2.203 | 89.330 | 9.451 |
|  |  | ${DS}_{HGLG}^{AP}$ | 77.978 | -116.787 | -0.065 | -0.659 | 201.540 | 14.196 |
|  |  | ${DS}_{HGLG}^{EF}$ | 100.908 | -71.016 | 0.211 | 0.749 | 138.400 | 11.764 |
|  |  | ST_HGLG_ | 95.959 | -116.781 | 0.424 | -0.167 | 263.170 | 16.223 |
|  |  | VO_HGLG_ | 135.086 | -76.821 | 0.669 | 1.053 | 233.095 | 15.267 |
|  |  | DS_HGLG_ | 77.486 | -86.891 | 0.768 | 0.802 | 139.767 | 11.822 |
|  | ***CO_2_*** | ${ST}_{HGLG}^{AP}$ | 18.966 | -14.155 | 0.040 | 0.442 | 8.501 | 2.916 |
|  |  | ${ST}_{HGLG}^{EF}$ | 18.344 | -11.613 | 0.093 | 1.660 | 3.960 | 1.990 |
|  |  | ${VO}_{HGLG}^{AP}$ | 19.798 | -21.933 | 0.028 | -0.207 | 13.501 | 3.674 |
|  |  | ${VO}_{HGLG}^{EF}$ | 20.199 | -20.235 | 0.009 | -0.256 | 8.974 | 2.996 |
|  |  | ${DS}_{HGLG}^{AP}$ | 23.544 | -29.492 | 0.052 | -0.347 | 19.948 | 4.466 |
|  |  | ${DS}_{HGLG}^{EF}$ | 22.335 | -21.933 | 0.145 | 0.362 | 11.426 | 3.380 |
|  |  | ST_HGLG_ | 29.891 | -19.685 | 0.219 | 0.680 | 25.436 | 5.043 |
|  |  | VO_HGLG_ | 23.164 | -24.160 | -0.086 | -0.582 | 21.618 | 4.650 |
|  |  | DS_HGLG_ | 31.278 | -15.841 | 0.175 | 1.636 | 11.635 | 3.411 |
|  | ***SO_2_*** | ${ST}_{HGLG}^{AP}$ | 5.854 | -6.566 | -0.068 | -0.304 | 1.579 | 1.257 |
|  |  | ${ST}_{HGLG}^{EF}$ | 9.917 | -4.625 | 0.084 | 2.354 | 0.829 | 0.911 |
|  |  | ${VO}_{HGLG}^{AP}$ | 14.070 | -6.405 | 0.088 | 0.653 | 3.176 | 1.782 |
|  |  | ${VO}_{HGLG}^{EF}$ | 8.794 | -6.441 | 0.010 | 0.823 | 1.543 | 1.242 |
|  |  | ${DS}_{HGLG}^{AP}$ | 10.754 | -11.517 | 0.031 | -0.101 | 3.779 | 1.944 |
|  |  | ${DS}_{HGLG}^{EF}$ | 8.408 | -5.989 | 0.066 | 0.627 | 2.288 | 1.513 |
|  |  | ST_HGLG_ | 18.064 | -7.762 | 0.060 | 1.228 | 4.782 | 2.187 |
|  |  | VO_HGLG_ | 14.926 | -9.738 | -0.003 | 0.384 | 3.293 | 1.815 |
|  |  | DS_HGLG_ | 12.467 | -6.788 | 0.011 | 0.680 | 2.231 | 1.494 |
| ***Validation*** | ***CS*** | ${ST}_{HGLG}^{AP}$ | 85.047 | -19.172 | 2.635 | 4.727 | 121.471 | 11.021 |
|  |  | ${ST}_{HGLG}^{EF}$ | 57.318 | -19.885 | 1.706 | 4.438 | 87.804 | 9.370 |
|  |  | ${VO}_{HGLG}^{AP}$ | 62.476 | -45.790 | 2.838 | 1.294 | 196.334 | 14.012 |
|  |  | ${VO}_{HGLG}^{EF}$ | 75.869 | -11.961 | 3.534 | 3.748 | 189.118 | 13.752 |
|  |  | ${DS}_{HGLG}^{AP}$ | 63.511 | -49.323 | 4.037 | 0.914 | 235.582 | 15.349 |
|  |  | ${DS}_{HGLG}^{EF}$ | 96.508 | -93.473 | 2.832 | 0.299 | 286.153 | 16.916 |
|  |  | ST_HGLG_ | 104.516 | -63.200 | 4.648 | 1.904 | 400.018 | 20.000 |
|  |  | VO_HGLG_ | 71.636 | -39.232 | 3.857 | 1.333 | 278.097 | 16.676 |
|  |  | DS_HGLG_ | 79.850 | -40.791 | 3.162 | 2.148 | 194.802 | 13.957 |
|  | ***CO_2_*** | ${ST}_{HGLG}^{AP}$ | 16.983 | -13.359 | 0.353 | 1.122 | 10.676 | 3.267 |
|  |  | ${ST}_{HGLG}^{EF}$ | 4.701 | -26.053 | -0.144 | -6.984 | 8.920 | 2.987 |
|  |  | ${VO}_{HGLG}^{AP}$ | 12.355 | -29.212 | -0.139 | -1.920 | 29.240 | 5.407 |
|  |  | ${VO}_{HGLG}^{EF}$ | 13.840 | -6.302 | 0.763 | 2.035 | 9.164 | 3.027 |
|  |  | ${DS}_{HGLG}^{AP}$ | 13.422 | -19.767 | 0.154 | -0.795 | 25.017 | 5.002 |
|  |  | ${DS}_{HGLG}^{EF}$ | 16.776 | -22.122 | 0.564 | -0.418 | 23.693 | 4.868 |
|  |  | ST_HGLG_ | 16.776 | -16.992 | 0.584 | 0.286 | 28.317 | 5.321 |
|  |  | VO_HGLG_ | 28.619 | -16.606 | 0.639 | 1.143 | 27.324 | 5.227 |
|  |  | DS_HGLG_ | 42.318 | -16.606 | 0.647 | 2.549 | 43.382 | 6.586 |
|  | ***SO_2_*** | ${ST}_{HGLG}^{AP}$ | 6.591 | -5.822 | 0.211 | 1.095 | 2.858 | 1.691 |
|  |  | ${ST}_{HGLG}^{EF}$ | 6.540 | -4.158 | 0.053 | 1.153 | 2.133 | 1.461 |
|  |  | ${VO}_{HGLG}^{AP}$ | 6.711 | -6.387 | -0.148 | 0.420 | 4.510 | 2.124 |
|  |  | ${VO}_{HGLG}^{EF}$ | 3.879 | -5.934 | -0.270 | -1.061 | 2.545 | 1.595 |
|  |  | ${DS}_{HGLG}^{AP}$ | 9.869 | -12.165 | -0.210 | -0.714 | 4.860 | 2.205 |
|  |  | ${DS}_{HGLG}^{EF}$ | 7.198 | -4.916 | 0.179 | 0.426 | 3.652 | 1.911 |
|  |  | ST_HGLG_ | 10.296 | -5.578 | 0.415 | 0.506 | 6.203 | 2.490 |
|  |  | VO_HGLG_ | 17.987 | -4.854 | 0.105 | 3.724 | 6.382 | 2.526 |
|  |  | DS_HGLG_ | 5.973 | -5.163 | 0.097 | 0.227 | 3.439 | 1.854 |
| ***Test*** | ***CS*** | ${ST}_{HGLG}^{AP}$ | 81.231 | -60.779 | 0.165 | 1.559 | 163.832 | 12.800 |
|  |  | ${ST}_{HGLG}^{EF}$ | 51.184 | -60.779 | -1.742 | -1.498 | 143.253 | 11.969 |
|  |  | ${VO}_{HGLG}^{AP}$ | 81.231 | -34.198 | 0.710 | 2.848 | 146.884 | 12.120 |
|  |  | ${VO}_{HGLG}^{EF}$ | 20.837 | -26.614 | 0.711 | -0.221 | 28.032 | 5.295 |
|  |  | ${DS}_{HGLG}^{AP}$ | 80.715 | -57.394 | 1.398 | 0.962 | 242.144 | 15.561 |
|  |  | ${DS}_{HGLG}^{EF}$ | 50.512 | -28.495 | 0.271 | 1.539 | 114.397 | 10.696 |
|  |  | ST_HGLG_ | 110.193 | -118.492 | 0.423 | -0.515 | 852.043 | 29.190 |
|  |  | VO_HGLG_ | 90.008 | -67.725 | 1.531 | 1.146 | 325.030 | 18.029 |
|  |  | DS_HGLG_ | 58.672 | -47.978 | 2.413 | 1.036 | 336.778 | 18.352 |
|  | ***CO_2_*** | ${ST}_{HGLG}^{AP}$ | 11.578 | -15.920 | 0.003 | -0.625 | 8.567 | 2.927 |
|  |  | ${ST}_{HGLG}^{EF}$ | 16.428 | -7.890 | 0.053 | 3.218 | 5.694 | 2.386 |
|  |  | ${VO}_{HGLG}^{AP}$ | 20.606 | -10.529 | -0.031 | 1.309 | 14.678 | 3.831 |
|  |  | ${VO}_{HGLG}^{EF}$ | 12.047 | -7.039 | -0.112 | 0.769 | 7.801 | 2.793 |
|  |  | ${DS}_{HGLG}^{AP}$ | 22.837 | -15.086 | -0.326 | 1.001 | 21.092 | 4.593 |
|  |  | ${DS}_{HGLG}^{EF}$ | 9.966 | -11.713 | -0.280 | -0.250 | 11.813 | 3.437 |
|  |  | ST_HGLG_ | 18.419 | -19.321 | 0.111 | 0.111 | 25.169 | 5.017 |
|  |  | VO_HGLG_ | 22.837 | -28.405 | -0.564 | -0.502 | 25.654 | 5.065 |
|  |  | DS_HGLG_ | 7.424 | -13.725 | -0.263 | -0.885 | 11.448 | 3.383 |
|  | ***SO_2_*** | ${ST}_{HGLG}^{AP}$ | 7.464 | -2.722 | 0.214 | 2.249 | 2.449 | 1.565 |
|  |  | ${ST}_{HGLG}^{EF}$ | 3.161 | -5.202 | -0.223 | -1.050 | 1.432 | 1.197 |
|  |  | ${VO}_{HGLG}^{AP}$ | 5.854 | -6.560 | -0.086 | -0.275 | 3.730 | 1.931 |
|  |  | ${VO}_{HGLG}^{EF}$ | 4.316 | -6.588 | -0.180 | -1.242 | 3.041 | 1.744 |
|  |  | ${DS}_{HGLG}^{AP}$ | 9.897 | -4.308 | 0.237 | 1.256 | 5.391 | 2.322 |
|  |  | ${DS}_{HGLG}^{EF}$ | 9.021 | -6.387 | -0.130 | 0.642 | 5.630 | 2.373 |
|  |  | ST_HGLG_ | 8.211 | -7.478 | -0.557 | 0.367 | 7.416 | 2.723 |
|  |  | VO_HGLG_ | 8.515 | -7.847 | -0.072 | 0.306 | 4.963 | 2.228 |
|  |  | DS_HGLG_ | 16.648 | -5.235 | 0.104 | 2.643 | 6.706 | 2.590 |
